# Melatonin attenuates reproductive toxicity of prothioconazole and its metabolite via spermatogenesis and oxidative stress

**DOI:** 10.1038/s42003-026-10311-y

**Published:** 2026-05-23

**Authors:** Jia-Hao Li, Jia-Li Liu, Ji-Hui Li, An-Ding Zhou, Hui Wang, Feng Qiu, Xiao-Li Xie, Qi Wang

**Affiliations:** 1 Guangzhou Key Laboratory of Forensic Multi-Omics for Precision Identification, School of Forensic Medicine, Southern Medical University, Guangzhou, China; 2 Guangzhou Women and Children’s Medical Center, National Children’s Medical Center for South Central Region, Guangzhou Medical University, Guangzhou, China; 3 Department of Laboratory Medicine, The Seventh Affiliated Hospital of Southern Medical University, Foshan, China; 4 Department of Toxicology, School of Public Health, Southern Medical University (Guangdong Provincial Key Laboratory of Tropical Disease Research), Guangzhou, China

**Keywords:** Agriculture, Endocrinology, Endocrine reproductive disorders

## Abstract

Prothioconazole (PTC), a widely used triazole fungicide, and its metabolite, prothioconazole-desthio (dPTC), have been frequently detected in environmental matrices, raising significant concerns regarding their potential adverse effects on mammalian reproductive health. While melatonin has demonstrated protective properties across multiple physiological systems, its efficacy in mitigating PTC- and dPTC-induced reproductive toxicity remains poorly understood. This study investigated the reproductive effects of PTC and dPTC in male mice and evaluated the potential protective effects of melatonin. PTC and dPTC exposure induced reproductive dysfunction, characterized by reduced sperm counts, disrupted spermatogenesis, altered HPG axis hormone secretion, elevated oxidative stress, and compromised BTB integrity. Melatonin co-treatment significantly attenuated these adverse effects, restoring sperm parameters, normalizing HPG axis function, mitigating oxidative stress, and preserving BTB integrity, possibly via the modulation of the MAPK/ERK signaling pathway. These findings underscore the potential reproductive toxicity of PTC/dPTC and propose melatonin as a promising therapeutic approach to alleviate the adverse reproductive impacts induced by triazole fungicides.

## Abstract (web summary)

Melatonin attenuates reproductive toxicity induced by PTC and dPTC in male mice via regulating HPG axis hormone secretion, enhancing spermatogenesis, modulating oxidative stress, and preserving BTB integrity.

## Graphical abstract

**Figure:**
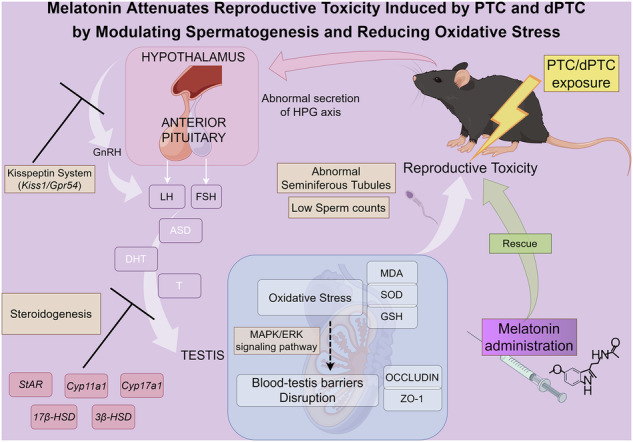

## Introduction

Prothioconazole (PTC), a triazole fungicide, is extensively used to control phytopathogenic fungi in cereals, wheat, and legume crops^1,2^. Both PTC and its metabolite, prothioconazole-desthio (dPTC), have been detected in terrestrial and aquatic environments^3,4^, raising environmental and human health safety concerns due to the potential for dietary and occupational exposure. Exposure to triazole fungicides can lead to a range of toxicities, including developmental^5,6^, hepatorenal^7^, and reproductive toxicity, as well as endocrine-disrupting effects^8^. Studies have demonstrated that PTC and dPTC induce endocrine disturbances in zebrafish^9^ and negatively impact sperm parameters in chickens by disrupting testosterone secretion and increasing oxidative stress levels^10^. Despite these findings, the reproductive toxicity of PTC and dPTC in mammals and potential preventative strategies remain largely unexplored.

The hypothalamic-pituitary-gonadal (HPG) axis is the cornerstone of reproductive regulation in mammals, and maintenance of steroid hormone homeostasis is essential for spermatogenesis. Pulsatile secretion of gonadotropin-releasing hormone (GnRH) by hypothalamic neurons triggers the release of luteinizing hormone (LH) and follicle-stimulating hormone (FSH) from the anterior pituitary, which in turn regulates testicular steroidogenesis and supports germ cell development^11^. Disruption of this hormonal cascade—particularly dysregulation of gonadotropins and testosterone—can impair Sertoli cell function and compromise the integrity of the blood–testis barrier (BTB), a specialized structure formed by tight junctions between adjacent Sertoli cells^12^. By limiting paracellular permeability, the BTB establishes a distinct microenvironment that supports post-meiotic germ cell development and sperm maturation^13^. In addition to endocrine control, the structural and functional integrity of the BTB is vulnerable to oxidative stress, which can disrupt tight junction proteins, induce germ cell apoptosis, and impair sperm maturation^14,15^. Maintaining an intact and functional BTB is essential for both the maintenance and re-initiation of spermatogenesis^16,17^.

Exposure to triazole fungicides has been shown to interfere with both steroidogenesis and redox homeostasis, suggesting their potential to disrupt multiple regulatory pathways essential for spermatogenesis. These compounds have been demonstrated to alter sex hormone synthesis^18^ and induce oxidative stress in various tissues^14,15^. Notably, both PTC and dPTC have been shown to cause oxidative stress-related damage in the liver and kidneys, indicating their ability to impair systemic redox balance and potentially affect testicular function^7^. However, the specific mechanisms by which PTC and dPTC disrupt male reproductive health—particularly through combined effects on the HPG axis, BTB integrity, and germ cell survival—remain poorly understood.

Melatonin (N-acetyl-5-methoxytryptamine, MT), an endogenous hormone secreted by the pineal gland, is well-established for its role in regulating circadian rhythms. Increasingly, research has focused on the protective effects of melatonin against reproductive toxicity^19,20^. As a potent antioxidant, melatonin can ameliorate reproductive toxicity induced by various toxicants by reducing oxidative stress^21,22^. Moreover, melatonin influences reproductive function by modulating steroid hormone secretion through its interactions with receptors and binding sites on the HPG axis^23^. However, the potential for melatonin to protect against the reproductive toxicity of the triazole fungicides PTC and dPTC has not yet been investigated.

Here, we aimed to investigate whether exposure to the triazole fungicide prothioconazole (PTC) and its metabolite prothioconazole-desthio (dPTC) impairs male reproductive function by disrupting the HPG axis and BTB integrity, and whether melatonin alleviates the reproductive toxicity caused by PTC and dPTC. We found that chronic PTC and dPTC exposure disrupted HPG axis hormone secretion, induced testicular oxidative stress, impaired BTB integrity, and led to spermatogenic dysfunction. Co-treatment with melatonin ameliorated these adverse effects by restoring redox homeostasis, improving spermatogenesis, and preserving BTB structure. Our study provides evidence that PTC and dPTC are reproductively toxic in male mammals and highlights melatonin as a potential therapeutic agent for mitigating the reproductive risks associated with triazole fungicide exposure.

## Results

### PTC and dPTC exposure results in reproductive toxicity in male mice

**Fig. 1:**
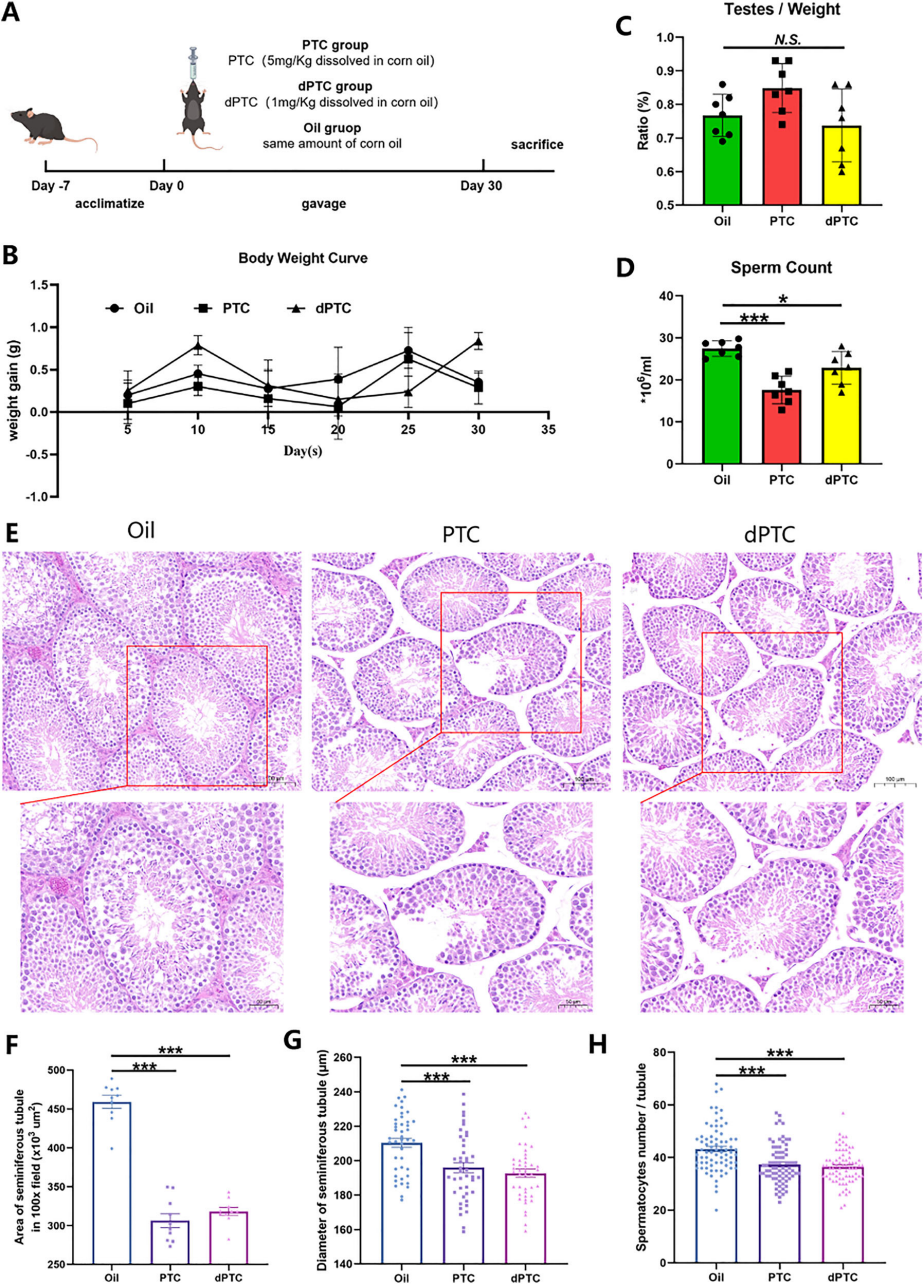
Exposure to PTC and dPTC induces reproductive toxicity in male mice. **A** The modeling flow chart of the PTC and dPTC exposure mouse models. ID: UARWI4816b. By Figdraw (https://www.figdraw.com). **B** Body weight change curve during modeling. *n* = 7 per group. **C** The testes-to-body weight ratio of each group. *n* = 7 per group. One-way ANOVA: *F*(2,18) = 3.315, *p* = 0.0595; Holm-Sidak’s multiple comparisons test: Oil vs. PTC, *p* = 0.1641; Oil vs. dPTC, *p* = 0.5115; **D** the sperm count of each group. *n* = 7 per group. One-way ANOVA: *F*(2,18) = 17.43, *p* < 0.0001; Holm-Sidak’s multiple comparisons test: Oil vs. PTC, *p* < 0.0001; Oil vs. dPTC, *p* = 0.0136. **E** Representative testicular tissue sections with H&E staining (scale bar: 100 μm), with magnified inset (scale bar: 50 μm). **F** The area of spermatogenic tubules (*n* = 10, One-way ANOVA: *F*(2,27) = 125.6, *p* < 0.0001; Holm-Sidak’s multiple comparisons test: Oil vs. PTC, *p* < 0.0001; Oil vs. dPTC, *p* < 0.0001), **G** the diameter of spermatogenic tubules (*n* = 43, One-way ANOVA: *F*(2,126) = 12.24, *p* < 0.0001; Holm-Sidak’s multiple comparisons test: Oil vs. PTC, *p* = 0.0002; Oil vs. dPTC, *p* < 0.0001), and **H** the number of spermatocytes per tubule in different groups (*n* = 75, One-way ANOVA: *F*(2,222) = 17.36, *p* < 0.0001; Holm-Sidak’s multiple comparisons test: Oil vs. PTC, *p* < 0.0001; Oil vs. dPTC, *p* < 0.0001). Two or three observation fields were randomly selected from each testicular H&E section. Data are expressed as mean ± SEM. *P < 0.05, **P < 0.01, ***P < 0.001. *N.S*. non-significant (*P* > 0.05).

To investigate the reproductive toxicity of PTC and dPTC, we established a chronic exposure model in male mice (Fig. 1A). Neither PTC nor dPTC exposure significantly affected body weight or the testes-to-body weight ratio (Fig. 1B, C). However, sperm counts were significantly reduced in both PTC- and dPTC-exposed mice compared to the control mice (Fig. 1D). Histological analysis of testicular tissues via hematoxylin and eosin (H&E) staining revealed structural abnormalities in the PTC and dPTC groups. Significant architectural disruption in the seminiferous tubules of PTC and dPTC groups compared to controls. Tubules exhibited disorganized germ cell layers and selective germ cell loss. (Fig. 1E). Furthermore, Exposure to PTC and dPTC resulted in a significant reduction in the area and diameter of seminiferous tubules, along with a decrease in the number of spermatocytes per tubule (Fig. 1F–H).

**Fig. 2:**
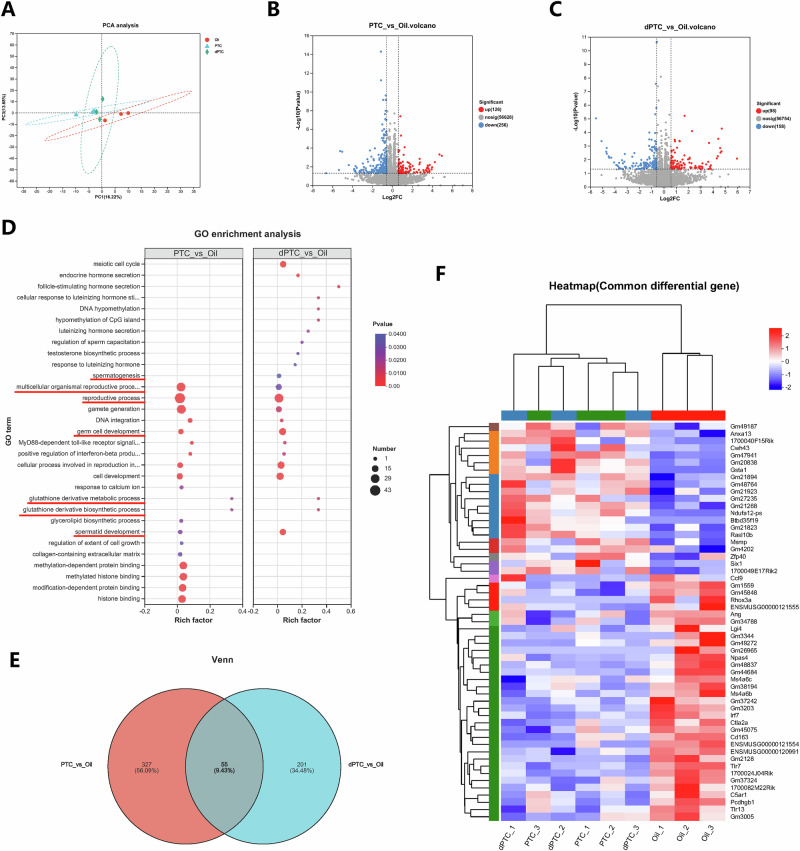
Transcriptomic changes in testicular tissue following PTC and dPTC exposure. **A** PCA plot analysis of testicular RNA-Seq. Red represents the Oil group, blue represents the PTC group, and green represents the dPTC group. *n* = 3 per group. **B** Volcano map of differential gene expression between the PTC group and the Oil group. **C** Volcano map of differential gene expression between the dPTC group and the Oil group. Red represents upregulated genes, blue represents downregulated genes, and white represents genes without significant change. *P* < 0.05 and fold change equal to or greater than 1.5. **D** Bubble plot of GO enrichment analysis of DEGs. **E** Venn diagram of DEGs. **F** Heatmap of common DEGs obtained by Venn analysis.

To explore the molecular mechanisms underlying PTC- and dPTC-induced reproductive toxicity, we performed RNA-Seq analysis on testicular tissues. Principal component analysis (PCA) demonstrated clear separation between the control (Oil) group and the PTC and dPTC groups, which clustered together (Fig. 2A). Compared with the control group, the PTC exposure group presented 382 differentially expressed genes (DEGs), with 126 upregulated and 256 downregulated (Fig. 2B). Similarly, dPTC exposure resulted in 256 DEGs, with 98 upregulated and 158 downregulated (Fig. 2C). Analysis of DEGs using GO pathway uncovered significant enrichment in pathways linked to reproductive processes, germ cell development, and spermatid development in both PTC and dPTC groups, suggesting impaired spermatogenesis (Fig. 2D). Additionally, pathways related to glutathione metabolism and biosynthesis were also enriched, indicating potential oxidative stress in the testes following exposure. Venn analysis identified 55 common DEGs between the PTC and dPTC groups (Fig. 2E). A heatmap of these common DEGs further highlighted the similar expression patterns in the PTC and dPTC groups, which differed substantially from those in the control group (Fig. 2F).

These results suggest that exposure to PTC and dPTC leads to reproductive toxicity in male mice, characterized by testicular structural abnormalities and abnormal gene expression associated with spermatogenesis and oxidative stress.

### PTC and dPTC exposure results in abnormalities in the HPG axis and spermatogenesis

**Fig. 3:**
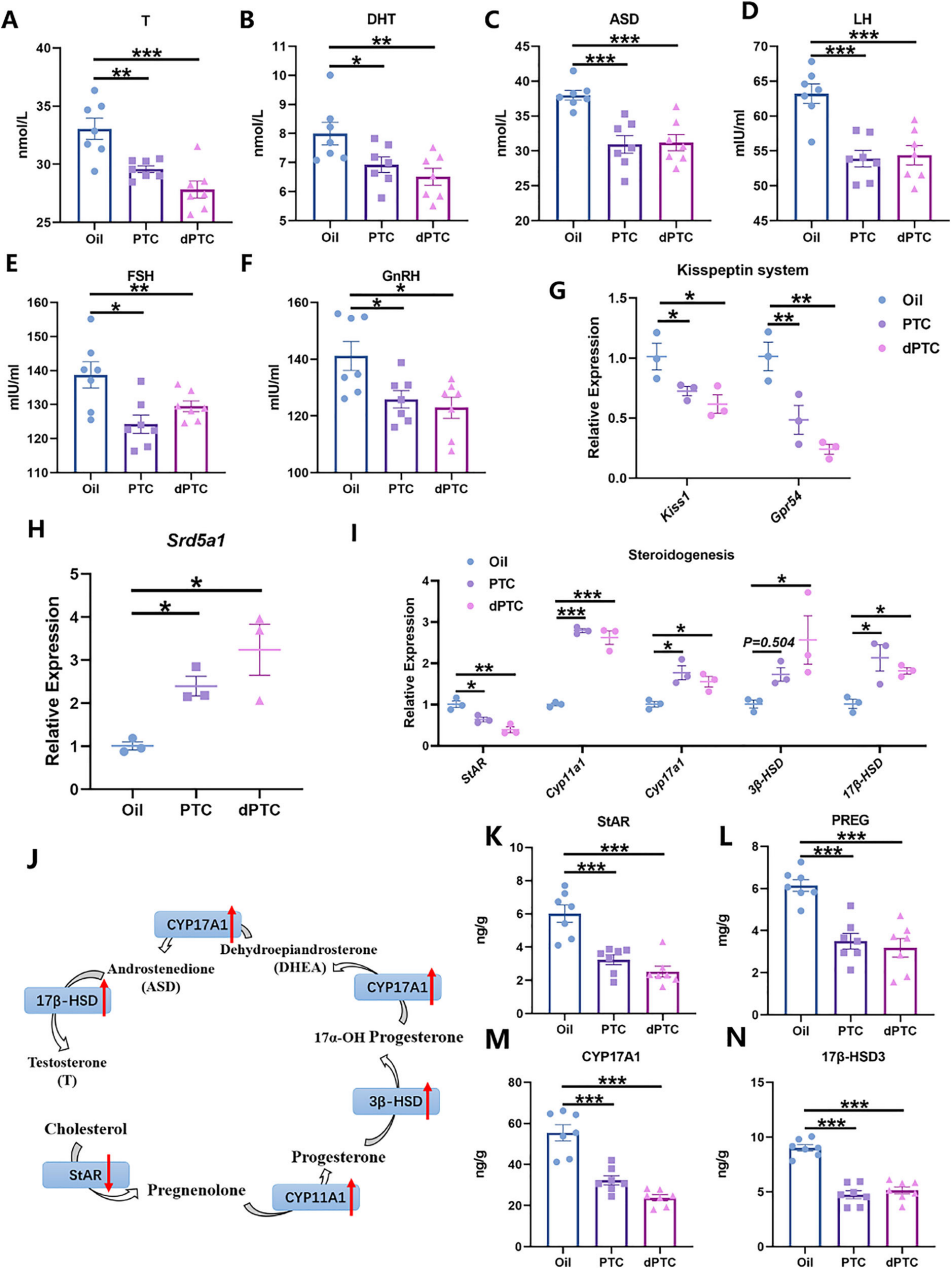
Exposure to PTC and dPTC induces abnormalities in the HPG axis and spermatogenesis. **A**–**F** The serum contents of T, DHT, ASD, LH, FSH, and GnRH were measured via ELISA. *n* = 7 per group. T: One-way ANOVA: *F*(2,18) = 14.44, *p* = 0.0002; Holm-Sidak’s multiple comparisons test: Oil vs. PTC, *p* = 0.0025; Oil vs. dPTC, *p* = 0.0001; DHT: One-way ANOVA: *F*(2,18) = 5.702, *p* = 0.0121; Holm-Sidak’s multiple comparisons test: Oil vs. PTC, *p* = 0.0300; Oil vs. dPTC, *p* = 0.0084; ASD: One-way ANOVA: *F*(2,18) = 13.71, *p* = 0.0002; Holm-Sidak’s multiple comparisons test: Oil vs. PTC, *p* = 0.0004; Oil vs. dPTC, *p* = 0.0004; FSH: One-way ANOVA: *F*(2,18) = 6.584, *p* = 0.0071; Holm-Sidak’s multiple comparisons test: Oil vs. PTC, *p* = 0.0042; Oil vs. dPTC, *p* = 0.0348; LH: One-way ANOVA: *F*(2,18) = 15.75, *p* = 0.0001; Holm-Sidak’s multiple comparisons test: Oil vs. PTC, *p* = 0.0002; Oil vs. dPTC, *p* = 0.0002; GnRH: One-way ANOVA: *F*(2,18) = 5.876, *p* = 0.0109; Holm-Sidak’s multiple comparisons test: Oil vs. PTC, *p* = 0.0154; Oil vs. dPTC, *p* = 0.0100. **G** The relative mRNA expression levels of *Kiss1* and *Gpr54* in different groups (*n* = 3). *Kiss1*: One-way ANOVA: *F*(2,6) = 6.367, *p* = 0.0329; Holm-Sidak’s multiple comparisons test: Oil vs. PTC, *p* = 0.0462; Oil vs. dPTC, *p* = 0.0270; *Gpr54*: One-way ANOVA: *F*(2,18) = 15.56, *p* = 0.0042; Holm-Sidak’s multiple comparisons test: Oil vs. PTC, *p* = 0.0097; Oil vs. dPTC, *p* = 0.0031. **H** The relative mRNA expression levels of *Srd5a1* (*n* = 3). One-way ANOVA: *F*(2,6) = 9.227, *p* = 0.0148; Holm-Sidak’s multiple comparisons test: Oil vs. PTC, *p* = 0.0385; Oil vs. dPTC, *p* = 0.0107; **I** the relative mRNA expression of steroidogenesis-related genes (*StAR, Cyp11a1, Cyp17a1, 3β-HSD, and 17β-HSD*) in different groups (*n* = 3). *StAR*: One-way ANOVA: *F*(2,6) = 19.30, *p* = 0.0024; Holm-Sidak’s multiple comparisons test: Oil vs. PTC, *p* = 0.0103; Oil vs. dPTC, *p* = 0.0017; *Cyp11a1*: One-way ANOVA: *F*(2,6) = 96.78, *p* < 0.0001; Holm-Sidak’s multiple comparisons test: Oil vs. PTC, *p* < 0.0001; Oil vs. dPTC, *p* < 0.0001; *Cyp17a1*: One-way ANOVA: *F*(2,6) = 9.491, *p* = 0.0139; Holm-Sidak’s multiple comparisons test: Oil vs. PTC, *p* = 0.0110; Oil vs. dPTC, *p* = 0.0233; *3β-HSD*: One-way ANOVA: *F*(2,6) = 4.754, *p* = 0.0579; Holm-Sidak’s multiple comparisons test: Oil vs. PTC, *p* = 0.5042; Oil vs. dPTC, *p* = 0.0428; *17β-HSD*: One-way ANOVA: *F*(2,6) = 8.281, *p* = 0.0188; Holm-Sidak’s multiple comparisons test: Oil vs. PTC, *p* = 0.0151; Oil vs. dPTC, *p* = 0.0297. **J** Changes of key enzymes involved in steroidogenesis induced by PTC and dPTC, and their roles in testosterone production. **K**–**N** The testicular contents of StAR, PREG, CYP17A1, and 17β-HSD3 were measured via ELISA. *n* = 7 per group. StAR: One-way ANOVA: *F*(2,18) = 22.00, *p* < 0.0001; Holm-Sidak’s multiple comparisons test: Oil vs. PTC, *p* < 0.0001; Oil vs. dPTC, *p* < 0.0001; PREG: *F*(2,18) = 19.43, *p* < 0.0001; Holm-Sidak’s multiple comparisons test: Oil vs. PTC, *p* < 0.0001; Oil vs. dPTC, *p* < 0.0001; CYP17A1: *F*(2,18) = 35.55, *p* < 0.0001; Holm-Sidak’s multiple comparisons test: Oil vs. PTC, *p* < 0.0001; Oil vs. dPTC, *p* < 0.0001; 17β-HSD3: *F*(2,18) = 51.42, *p* < 0.0001; Holm-Sidak’s multiple comparisons test: Oil vs. PTC, *p* < 0.0001; Oil vs. dPTC, *p* < 0.0001. Data are expressed as mean ± SEM. *P < 0.05, **P < 0.01, ***P < 0.001. *N.S*. non-significant (*P* > 0.05).

Given the pivotal role of the HPG axis in regulating reproductive function, we assessed the serum concentrations of HPG-axis steroid hormones. In comparison to the control group, both PTC and dPTC exposure significantly reduced serum levels of T, DHT, and ASD (Fig. 3A–C). Concurrently, upstream regulatory hormones were consistently suppressed. Both treatment groups exhibited significant reductions in LH and FSH (Fig. 3D, E), as well as in GnRH (Fig. 3F).

Disrupted HPG axis hormone secretion, coupled with functional enrichment analysis of the DEGs, suggested impaired spermatogenesis. Androgens are essential for spermatogenesis, and their observed reduction following PTC and dPTC exposure further supports this hypothesis. To investigate the underlying molecular mechanisms, we examined genes involved in HPG axis hormone secretion and steroidogenesis. In hypothalamic tissue, the mRNA expression levels of *Kiss1* and *Gpr54*, components of the kisspeptin system crucial for HPG axis regulation, were significantly downregulated in both the PTC and dPTC groups (Fig. 3G). In testicular tissue, we observed a significant upregulation of *Srd5a1*, a key enzyme in androgen synthesis, following PTC and dPTC exposure (Fig. 3H). Furthermore, the expression of steroidogenesis-related genes in testicular tissue was also altered: *StAR* expression was significantly downregulated, whereas *Cyp11a1*, *Cyp17a1*, *17β-HSD*, and *3β-HSD* expression was significantly upregulated in both treatment groups (Fig. 3I, J). To further investigate the mechanisms underlying the reduced sex hormone levels, we quantified the testicular protein content of key steroidogenic enzymes (StAR, CYP17A1, 17β-HSD3) and a precursor (pregnenolone, PREG) by ELISA. In contrast to the observed mRNA upregulation for some targets, the protein levels of StAR, PREG, CYP17A1, and 17β-HSD3 were all significantly decreased following PTC and dPTC exposure (Fig. 3K–N). The discordance between increased mRNA expression and decreased protein content for CYP17A1 and 17β-HSD3 may suggest the involvement of post-transcriptional regulation. These findings collectively indicate that PTC and dPTC exposure disrupts HPG axis hormone secretion and alters the expression and translation of genes involved in androgen synthesis, potentially leading to impaired spermatogenesis.

### PTC and dPTC exposure disrupts the BTB and induces testicular oxidative stress

**Fig. 4:**
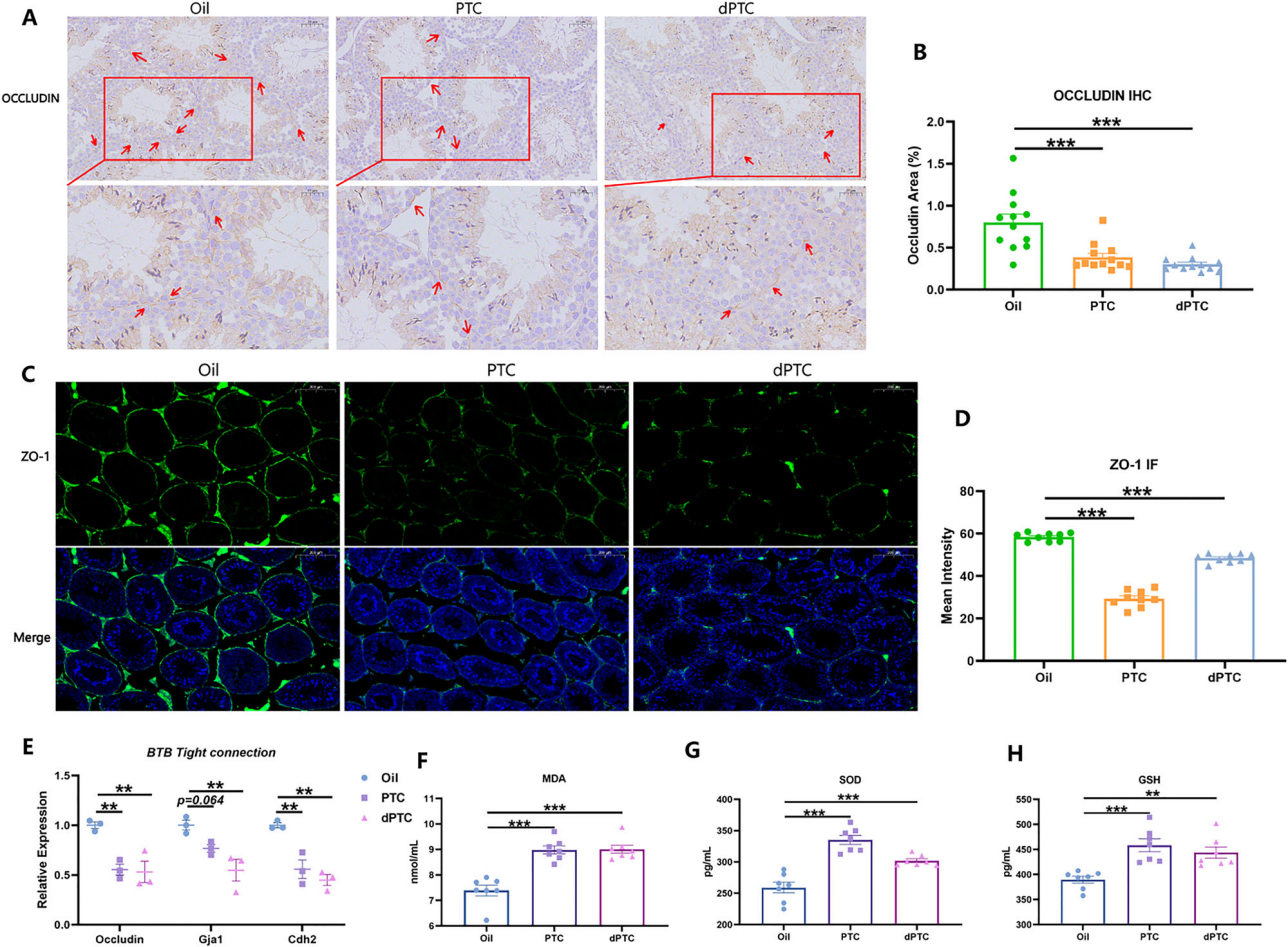
Exposure to PTC and dPTC disrupts the BTB and induces testicular oxidative stress. **A** Representative images of OCCLUDIN IHC staining. Red arrows indicate the OCCLUDIN positive staining area. Scale bar, 100 μm. **B** Statistical diagrams of the percentage of the OCCLUDIN-positive area (*n* = 12). Welch’s ANOVA test: *W*(2,19.51) = 11.94, *p* = 0.0004; Dunnett’s T3 multiple comparisons test: Oil vs. PTC, *p* = 0.0030; Oil vs. dPTC, *p* = 0.0011. Two or three observation fields were randomly selected from each testicular IHC section for statistical analysis. **C** Representative images of ZO-1 IF staining (in green). Nuclei were labeled with DAPI (in blue). Scale bar, 200 μm. **D** Statistical diagrams of ZO-1 mean fluorescence intensity (*n* = 9). One-way ANOVA: *F*(2,24) = 250.9, *p* < 0.0001; Holm-Sidak’s multiple comparisons test: Oil vs. PTC, *p* < 0.0001; Oil vs. dPTC, *p* < 0.0001. Two or three observation fields were randomly selected from each testicular IF section for statistical analysis. **E** Relative mRNA expression of tight junction proteins (*Occludin*, *Gja1*, and *Cdh2*) in different groups (*n* = 3). *Occludin*: One-way ANOVA: *F*(2,6) = 13.74, *p* = 0.0058; Holm-Sidak’s multiple comparisons test: Oil vs. PTC, *p* = 0.0079; Oil vs. dPTC, *p* = 0.0063; *Gja1*: One-way ANOVA: *F*(2,6) = 9.758, *p* = 0.0130; Holm-Sidak’s multiple comparisons test: Oil vs. PTC, *p* = 0.0635; Oil vs. dPTC, *p* = 0.0090; *Cdh2*: One-way ANOVA: *F*(2,6) = 21.00, *p* = 0.0020; Holm-Sidak’s multiple comparisons test: Oil vs. PTC, *p* = 0.0027; Oil vs. dPTC, *p* = 0.0017. **F**–**H** The contents of MDA, SOD, and GSH in testicular tissue were measured by ELISA. *n* = 7 per group. MDA: One-way ANOVA: *F*(2,18) = 27.33, *p* < 0.0001; Holm-Sidak’s multiple comparisons test: Oil vs. PTC, *p* < 0.0001; Oil vs. dPTC, *p* < 0.0001; SOD: One-way ANOVA: *F*(2,18) = 32.00, *p* < 0.0001; Holm-Sidak’s multiple comparisons test: Oil vs. PTC, *p* < 0.0001; Oil vs. dPTC, *p* = 0.0003; GSH: One-way ANOVA: *F*(2,18) = 11.53, *p* = 0.0006; Holm-Sidak's multiple comparisons test: Oil vs. PTC, *p* = 0.0005; Oil vs. dPTC, *p* = 0.0021. Data are expressed as mean ± SEM. *P < 0.05, **P < 0.01, ***P < 0.001. *N.S*. non-significant (*P* > 0.05).

The BTB is essential for maintaining the testicular microenvironment. To assess BTB integrity, we examined the expression of tight junction proteins. IHC revealed a significant reduction in OCCLUDIN protein expression in testicular tissue following PTC and dPTC exposure (Fig. 4A, B). Similarly, IF analysis revealed a significant decrease in ZO-1 protein expression (Fig. 4C, D). Furthermore, the mRNA expression levels of the tight junction genes *Occludin*, *Gja1*, and *Cdh2* were also significantly downregulated (Fig. 4E). These results indicate that PTC and dPTC exposure disrupts BTB integrity.

To evaluate the impact of PTC and dPTC on testicular oxidative stress, we measured the MDA, SOD, and GSH levels in testicular tissue via ELISA kits. Compared to the control group, both PTC and dPTC exposure significantly increased MDA, SOD, and GSH levels (Fig. 4F–H), indicating the induction of testicular oxidative stress.

### Melatonin alleviates PTC- and dPTC-induced reproductive toxicity

**Fig. 5:**
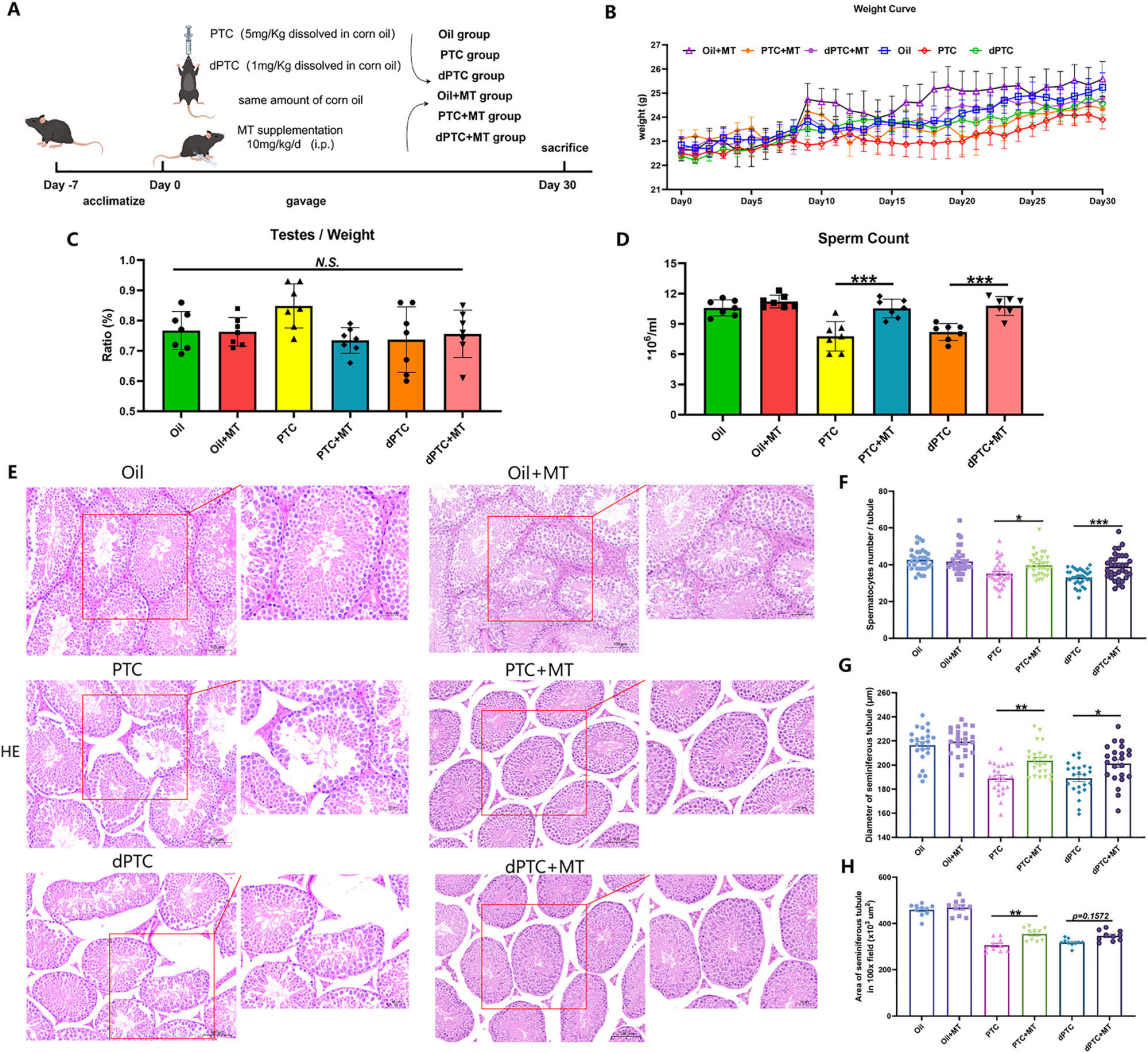
Melatonin alleviates reproductive toxicity induced by PTC and dPTC. **A** The modeling flow chart of the melatonin supplementation mouse model. ID: AOUAYbd18c. By Figdraw (https://www.figdraw.com). **B** Body weight curves of each group. *n* = 7 per group. **C** The testes-to-body weight ratio of each group. *n* = 7 per group. One-way ANOVA: *F*(5,36) = 2.352, *p* = 0.0603; Tukey’s post hoc test: Oil vs. PTC, *p* = 0.3051; Oil vs. dPTC, *p* = 0.9696; PTC vs. PTC + MT, *p* = 0.0558; dPTC vs. dPTC+MT, *p* = 0.9965. **D** The sperm count of each group. *n* = 7 per group. One-way ANOVA: *F*(5,36) = 16.34, *p* < 0.0001; Tukey’s post hoc test: Oil vs. PTC, *p* < 0.0001; Oil vs. dPTC, *p* = 0.0006; PTC vs. PTC + MT, *p* < 0.0001; dPTC vs. dPTC+MT, *p* = 0.0002. **E** Representative testicular tissue sections with H&E staining (scale bar: 100 μm), with magnified inset (scale bar: 50 μm). **F** The number of spermatocytes per tubule (*n* = 33, One-way ANOVA: *F*(5,192) = 12.39, *p* < 0.0001; Tukey’s post hoc test: Oil vs. PTC, *p* < 0.0001; Oil vs. dPTC, *p* < 0.0001; PTC vs. PTC + MT, *p* = 0.0284; dPTC vs. dPTC+MT, *p* = 0.0025), **G** the diameter of spermatogenic tubules (*n* = 23, One-way ANOVA: *F*(5,132) = 20.86, *p* < 0.0001; Tukey’s post hoc test: Oil vs. PTC, *p* < 0.0001; Oil vs. dPTC, *p* < 0.0001; PTC vs. PTC + MT, *p* = 0.0046; dPTC vs. dPTC+MT, *p* = 0.0344), and **H** the percentage of the spermatogenic tubule area (*n* = 10, One-way ANOVA: *F*(5,54) = 72.88, *p* < 0.0001; Tukey’s post hoc test: Oil vs. PTC, *p* < 0.0001; Oil vs. dPTC, *p* < 0.0001; PTC vs. PTC + MT, *p* = 0.0016; dPTC vs. dPTC+MT, *p* = 0.1572). Two or three observation fields were randomly selected from each testicular H&E section. Data are expressed as mean ± SEM. *P < 0.05, **P < 0.01, ***P < 0.001. *N.S*. non-significant (*P* > 0.05).

While the protective effects of melatonin on reproductive function have been documented, its impact on PTC- and dPTC-induced reproductive toxicity remains unexplored. To address this, we established PTC and dPTC exposure mouse models, as described previously, and concurrently administered melatonin to assess its potential ameliorative effects (Fig. 5A). Melatonin supplementation did not significantly affect body weight or the testes-to-body weight ratio in either the exposed or the control groups (Fig. 5B, C). However, it reversed the reduction in sperm counts observed in PTC- and dPTC-exposed mice (Fig. 5D). Histological examination of testicular tissue revealed that melatonin restored the normal architecture of seminiferous tubules (Fig. 5E). This restoration was accompanied by significant increases in the number of spermatocytes per tubule and the diameter of the seminiferous tubules (Fig. 5F, G). Although melatonin supplementation appeared to boost the percentage of the spermatogenic tubule area, this effect did not achieve statistical significance in the dPTC+MT group relative to the dPTC group alone (Fig. 5H).

### Melatonin rescues HPG axis function and spermatogenesis in PTC- and dPTC-exposed mice

**Fig. 6:**
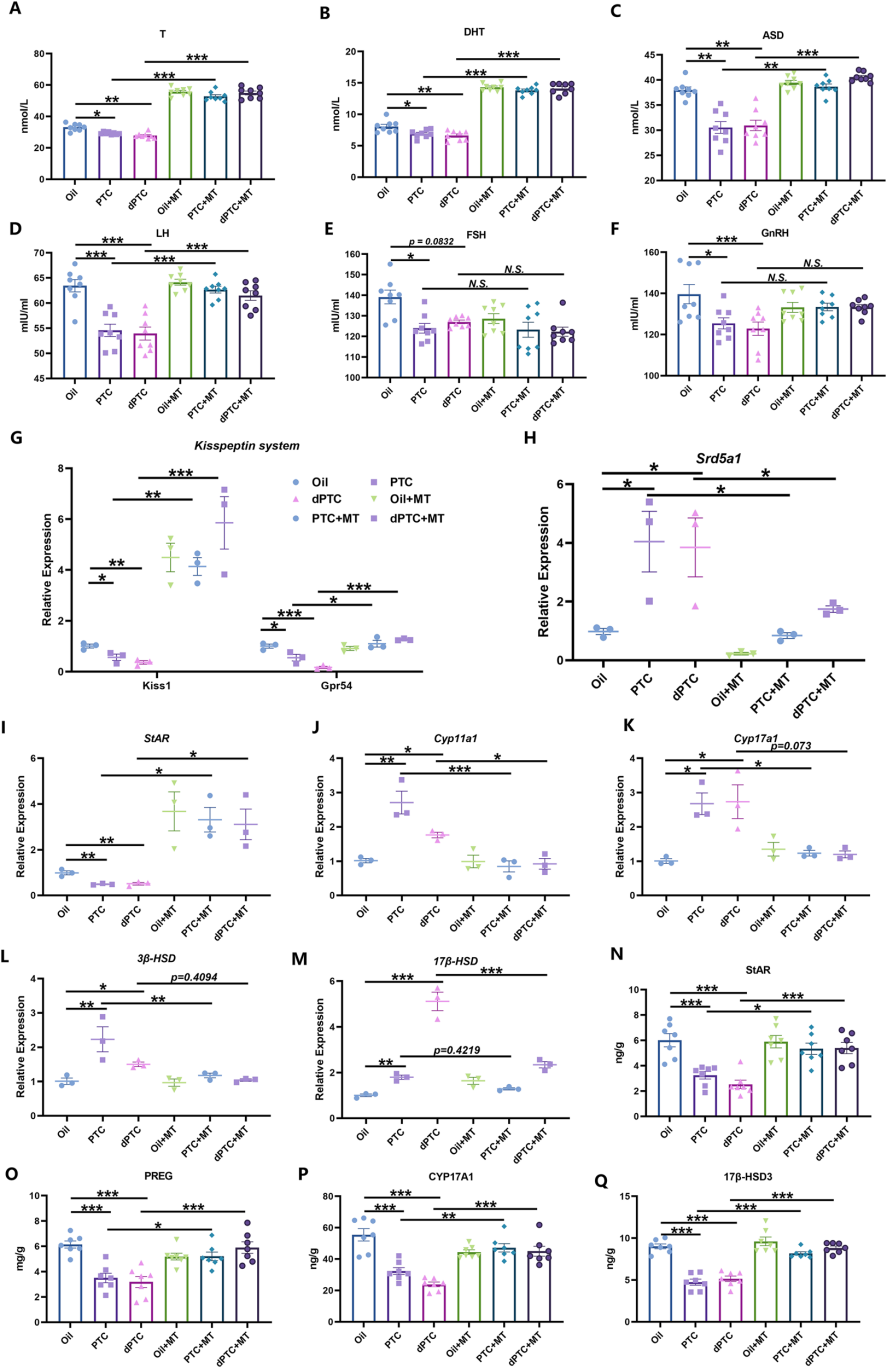
Melatonin restores HPG axis function and spermatogenesis in mice exposed to PTC and dPTC. **A**–**F** The serum contents of T, DHT, ASD, LH, FSH, and GnRH were measured by ELISA. *n* = 8 per group. T: One-way ANOVA: *F*(5,42) = 243.2, *p* < 0.0001; Tukey’s post hoc test: Oil vs. PTC, *p* = 0.0373; Oil vs. dPTC, *p* = 0.0012; PTC vs. PTC + MT, *p* < 0.0001; dPTC vs. dPTC+MT, *p* < 0.0001. DHT: One-way ANOVA: *F*(5,42) = 207.1, *p* < 0.0001; Tukey’s post hoc test: Oil vs. PTC, *p* = 0.0389; Oil vs. dPTC, *p* = 0.0045; PTC vs. PTC + MT, *p* < 0.0001; dPTC vs. dPTC+MT, *p* < 0.0001. ASD: Welch’s ANOVA *W*(5,19.12) = 24.34, *p* < 0.0001; Dunnett’s T3 multiple comparisons test: Oil vs. PTC, *p* = 0.0027; Oil vs. dPTC, *p* = 0.0015; PTC vs. PTC + MT, *p* = 0.0179; dPTC vs. dPTC+MT, *p* < 0.0001. FSH: Welch’s ANOVA *W*(5,18.36) = 3.665, *p* = 0.0005; Dunnett’s T3 multiple comparisons test: Oil vs. PTC, *p* = 0.0328; Oil vs. dPTC, *p* = 0.0832; PTC vs. PTC + MT, *p* > 0.9999; dPTC vs. dPTC+MT, *p* = 0.5460. LH: One-way ANOVA: *F*(5,42) = 19.89, *p* < 0.0001; Tukey’s post hoc test: Oil vs. PTC, *p* < 0.0001; Oil vs. dPTC, *p* < 0.0001; PTC vs. PTC + MT, *p* < 0.0001; dPTC vs. dPTC+MT, *p* < 0.0001. GnRH: One-way ANOVA: *F*(5,42) = 4.495, *p* = 0.0023; Tukey’s post hoc test: Oil vs. PTC, *p* = 0.0143; Oil vs. dPTC, *p* = 0.0023; PTC vs. PTC + MT, *p* = 0.3870; dPTC vs. dPTC+MT, *p* = 0.1218. **G** The relative mRNA expression levels of *Kiss1* and *Gpr54* in different groups (*n* = 3). *Kiss1*: One-way ANOVA: *F*(5,12) = 21.98, *p* < 0.0001; Tukey’s post hoc test: Oil vs. PTC, *p* = 0.0372; Oil vs. dPTC, *p* = 0.0070; PTC vs. PTC + MT, *p* = 0.0023; dPTC vs. dPTC+MT, *p* < 0.0001. *Gpr54*: One-way ANOVA: *F*(5,12) = 19.86, *p* < 0.0001; Tukey’s post hoc test: Oil vs. PTC, *p* = 0.0356; Oil vs. dPTC, *p* = 0.0003; PTC vs. PTC + MT, *p* = 0.0105; dPTC vs. dPTC+MT, *p* < 0.0001. **H** The relative mRNA expression levels of *Srd5a1* in different groups (*n* = 3). One-way ANOVA: *F*(5,12) = 7.511, *p* = 0.0021; Tukey’s post hoc test: Oil vs. PTC, *p* = 0.0303; Oil vs. dPTC, *p* = 0.0452; PTC vs. PTC + MT, *p* = 0.0229; dPTC vs. dPTC+MT, *p* = 0.0341. **I**–**M** The relative mRNA expression of steroidogenesis-related genes *(StAR, Cyp11a1, Cyp17a1, 3β-HSD*, and *17β-HSD*) in different groups (*n* = 3). *StAR*: One-way ANOVA: *F*(5,12) = 15.85, *p* < 0.0001; Tukey’s post hoc test: Oil vs. PTC, *p* = 0.0030; Oil vs. dPTC, *p* = 0.0040; PTC vs. PTC + MT, *p* = 0.0168; dPTC vs. dPTC+MT, *p* = 0.0289. *Cyp11a1*: One-way ANOVA: *F*(5,12) = 19.86, *p* < 0.0001; Tukey’s post hoc test: Oil vs. PTC, *p* = 0.0027; Oil vs. dPTC, *p* = 0.0433; PTC vs. PTC + MT, *p* = 0.0001; dPTC vs. dPTC+MT, *p* = 0.0466. *Cyp17a1*: One-way ANOVA: *F*(5,12) = 6.373, *p* = 0.0041; Tukey’s post hoc test: Oil vs. PTC, *p* = 0.0148; Oil vs. dPTC, *p* = 0.0380; PTC vs. PTC + MT, *p* = 0.0324; dPTC vs. dPTC+MT, *p* = 0.0731. *3β-HSD*: One-way ANOVA: *F*(5,12) = 8.747, *p* = 0.0011; Tukey’s post hoc test: Oil vs. PTC, *p* = 0.0021; Oil vs. dPTC, *p* = 0.0121; PTC vs. PTC + MT, *p* = 0.0069; dPTC vs. dPTC+MT, *p* = 0.4094. *17β-HSD*: One-way ANOVA: *F*(5,12) = 66.70, *p* < 0.0001; Tukey’s post hoc test: Oil vs. PTC, *p* = 0.0019; Oil vs. dPTC, *p* < 0.0001; PTC vs. PTC + MT, *p* = 0.4219; dPTC vs. dPTC+MT, *p* < 0.0001. **N**–**Q** The testicular contents of StAR, PREG, CYP17A1, and 17β-HSD3 were measured via ELISA. *n* = 7 per group. StAR: One-way ANOVA: *F*(5,36) = 11.96, *p* < 0.0001; Tukey’s post hoc test: Oil vs. PTC, *p* = 0.0007; Oil vs. dPTC, *p* < 0.0001; PTC vs. PTC + MT, *p* = 0.0155; dPTC vs. dPTC+MT, *p* = 0.0004; PREG: One-way ANOVA: *F*(5,36) = 11.41, *p* < 0.0001; Tukey’s post hoc test: Oil vs. PTC, *p* = 0.0001; Oil vs. dPTC, *p* < 0.0001; PTC vs. PTC + MT, *p* = 0.0241; dPTC vs. dPTC+MT, *p* < 0.0001; CYP17A1: One-way ANOVA: *F*(5,36) = 18.52, *p* < 0.0001; Tukey’s post hoc test: Oil vs. PTC, *p* < 0.0001; Oil vs. dPTC, *p* < 0.0001; PTC vs. PTC + MT, *p* = 0.0040; dPTC vs. dPTC+MT, *p* < 0.0001; 17β-HSD3: One-way ANOVA: *F*(5,36) = 38.42, *p* < 0.0001; Tukey’s post hoc test: Oil vs. PTC, *p* < 0.0001; Oil vs. dPTC, *p* < 0.0001; PTC vs. PTC + MT, *p* < 0.0001; dPTC vs. dPTC+MT, *p* < 0.0001. Data are expressed as mean ± SEM. *P < 0.05, **P < 0.01, ***P < 0.001. *N.S*. non-significant (*P* > 0.05).

We next investigated the effects of melatonin on HPG axis hormone secretion and the gene expression of androgen synthesis and HPG axis regulation. Melatonin supplementation significantly increased the serum levels of T, DHT, and ASD in PTC- and dPTC-exposed mice, restoring them to levels comparable to those in the control group (Fig. 6A–C). Similarly, melatonin reversed the dysregulation of LH secretion (Fig. 6D). However, no significant improvement was observed in FSH (Fig. 6E). Although GnRH levels showed a tendency to increase following melatonin treatment, this change did not reach statistical significance compared to the PTC and dPTC groups (Fig. 6F). In hypothalamic tissue, melatonin reversed the PTC- and dPTC-induced downregulation of *Kiss1* and *Gpr54* expression (Fig. 6G). The aberrant upregulation of *Srd5a1* in testicular tissue was also mitigated by melatonin supplementation (Fig. 6H). Furthermore, melatonin normalized the expression of the steroidogenesis-related genes *StAR*, *Cyp11a1*, *Cyp17a1*, *3β-HSD*, and *17β-HSD* to baseline levels (Fig. 6I–M). Critically, at the protein level, melatonin treatment effectively rescued the decreased testicular content of key steroidogenic factors—StAR, pregnenolone (PREG), CYP17A1, and 17β-HSD3—that were observed after PTC and dPTC exposure (Fig. 6N–Q). This restoration of protein levels, alongside the normalization of their corresponding mRNA expression, confirms that melatonin comprehensively rectifies the disrupted steroidogenic pathway. These findings demonstrate that melatonin rescues HPG axis function and spermatogenesis impaired by PTC and dPTC exposure.

### Melatonin restores BTB integrity and attenuates testicular oxidative stress in PTC- and dPTC-exposed mice

**Fig. 7:**
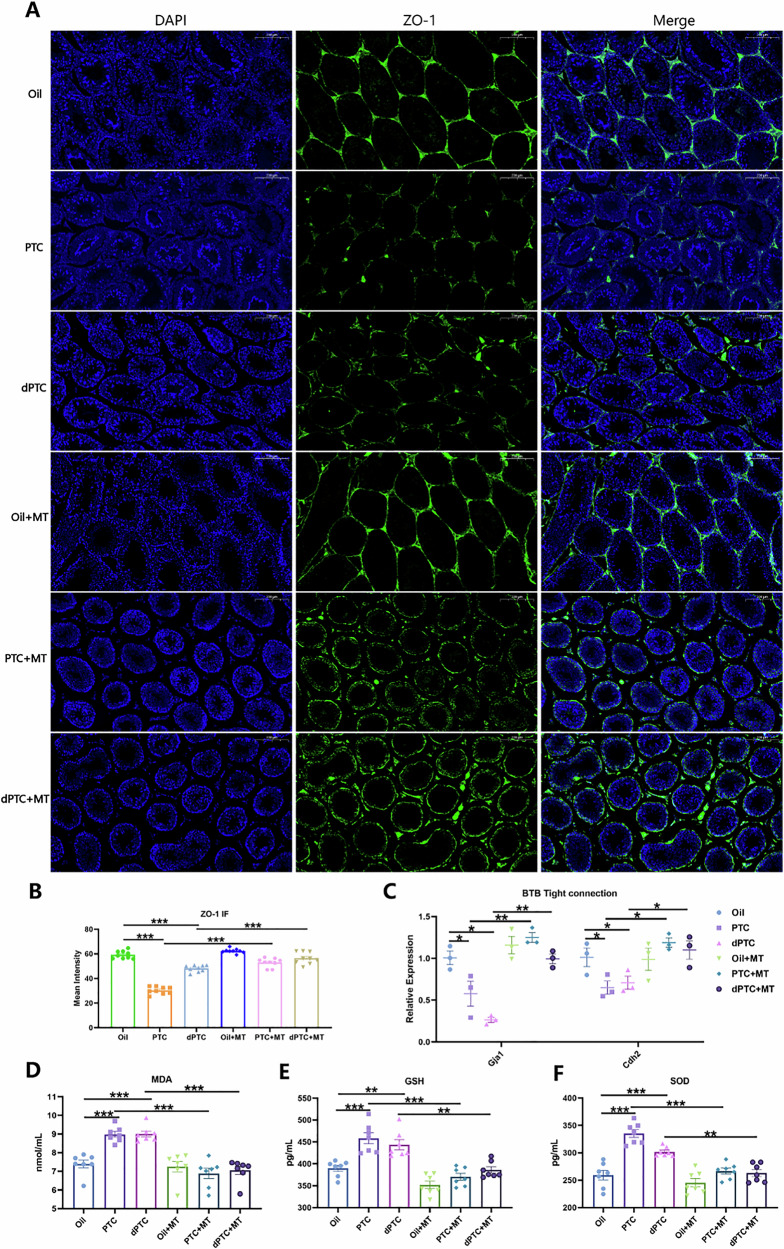
Melatonin improves BTB impairment and ameliorates testicular oxidative stress damage in PTC- and dPTC-exposed mice. **A** Representative images of ZO-1 IF staining (in green) in each group. Nuclei were labeled with DAPI (in blue). Scale bar, 200 μm. **B** Statistical diagrams of ZO-1 mean fluorescence intensity in different groups (*n* = 9). One-way ANOVA: *F*(5,48) = 110.3, *p* < 0.0001; Tukey’s post hoc test: Oil vs. PTC, *p* < 0.0001; Oil vs. dPTC, *p* < 0.0001; PTC vs. PTC + MT, *p* < 0.0001; dPTC vs. dPTC+MT, *p* < 0.0001. Two or three observation fields were randomly selected from each testicular IF section for statistical analysis. **C** Relative mRNA expression of tight junction proteins (*Gja1 and Cdh2*) in different groups (*n* = 3). **D**–**F** The contents of MDA, GSH, and SOD in testicular tissue were measured by ELISA. *n* = 7 per group. MDA: One-way ANOVA: *F*(5,36) = 18.97, *p* < 0.0001; Tukey’s post hoc test: Oil vs. PTC, *p* = 0.0002; Oil vs. dPTC, *p* = 0.0001; PTC vs. PTC + MT, *p* < 0.0001; dPTC vs. dPTC+MT, *p* < 0.0001; GSH: One-way ANOVA: *F*(5,36) = 20.06, *p* < 0.0001; Tukey’s post hoc test: Oil vs. PTC, *p* = 0.0001; Oil vs. dPTC, *p* = 0.0031; PTC vs. PTC + MT, *p* < 0.0001; dPTC vs. dPTC+MT, *p* = 0.0015; SOD: One-way ANOVA: *F*(5,36) = 24.34, *p* < 0.0001; Tukey’s post hoc test: Oil vs. PTC, *p* < 0.0001; Oil vs. dPTC, *p* = 0.0007; PTC vs. PTC + MT, *p* < 0.0001; dPTC vs. dPTC+MT, *p* = 0.1068. Data are expressed as mean ± SEM. *P < 0.05, **P < 0.01, ***P < 0.001. *N.S*. non-significant (*P* > 0.05).

We then investigated the effects of melatonin on BTB integrity and testicular oxidative stress. IF analysis revealed that melatonin supplementation restored ZO-1 protein expression to control levels, reversing the reduction induced by PTC and dPTC exposure (Fig. 7A, B). The expression levels of the tight junction genes *Gja1* and *Cdh2* were also significantly upregulated by melatonin (Fig. 7C), indicating that melatonin restores BTB integrity. Moreover, melatonin significantly decreased the MDA, GSH, and SOD levels in testicular tissue, effectively attenuating the oxidative stress induced by PTC and dPTC (Fig. 7D–F).

These findings demonstrate that melatonin protects against PTC- and dPTC-induced male reproductive toxicity by rescuing HPG axis function and spermatogenesis, restoring BTB integrity, and attenuating testicular oxidative stress.

### MAPK/ERK signaling pathway implicates in reproductive toxicity induced by PTC and dPTC

**Fig. 8:**
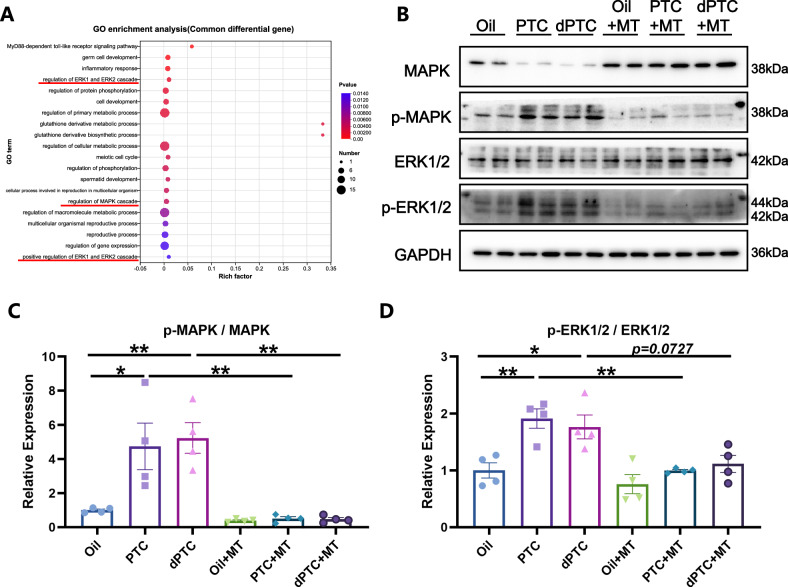
MAPK/ERK signaling pathway involvement in reproductive toxicity induced by PTC and dPTC. **A** Bubble plot of GO enrichment analysis of common DEGs obtained by Venn analysis. **B** Protein expression levels of MAPK, p-MAPK, ERK1/2, p-ERK1/2, and GAPDH. **C** Results of semi-quantitative analysis of p-MAPK/MAPK (*n* = 4, One-way ANOVA: *F*(5,18) = 11.58, *p* < 0.0001; Tukey’s post hoc test: Oil vs. PTC, *p* = 0.0104; Oil vs. dPTC, *p* = 0.0035; PTC vs. PTC + MT, *p* = 0.0035; dPTC vs. dPTC+MT, *p* = 0.0011) and **D** p-ERK1/2/ERK1/2 (*n* = 4, One-way ANOVA: *F*(5,18) = 9.214, *p* = 0.0002; Tukey’s post hoc test: Oil vs. PTC, *p* = 0.0061; Oil vs. dPTC, *p* = 0.0252; PTC vs. PTC + MT, *p* = 0.0059; dPTC vs. dPTC+MT, *p* = 0.0727). GAPDH served as a loading control, and the data were normalized to GAPDH. The original protein band is shown in the Supplementary Data 3. Data are expressed as mean ± SEM. *P < 0.05, **P < 0.01, ***P < 0.001. *N.S*. non-significant (*P* > 0.05).

To explore the potential molecular mechanisms underlying PTC- and dPTC-induced reproductive toxicity, we performed GO enrichment analysis of DEGs to both PTC and dPTC exposure groups in testicular tissue. The analysis uncovered notable enrichment in pathways linked to the MAPK and ERK signaling pathways (Fig. 8A). We further investigated the activation status of the MAPK/ERK signaling pathway via Western blot. PTC and dPTC exposure significantly increased the ratios of phosphorylated MAPK (p-MAPK) to total MAPK and phosphorylated ERK1/2 (p-ERK1/2) to total ERK1/2, suggesting the activation of this pathway. Melatonin supplementation attenuated this increase, restoring the p-MAPK/MAPK and p-ERK1/2/ERK1/2 ratios toward control levels (Fig. 8B–D). Our findings suggest that the MAPK/ERK signaling pathway may play a role in PTC- and dPTC-induced reproductive toxicity, and that the protective effects of melatonin could involve modulating this pathway. Further studies are necessary to definitively establish the role of MAPK/ERK signaling and explore the underlying mechanisms involved.

## Discussion

PTC, a widely used agricultural triazole fungicide, and its desulfurated metabolite dPTC have been detected in environmental matrices, raising concerns regarding their reproductive toxicity in both males and females^24–26^. The potential of melatonin, a potent antioxidant, to mitigate PTC/dPTC-induced reproductive toxicity remains to be elucidated. This study investigated the reproductive toxicity of PTC and dPTC in male mammals and demonstrated that melatonin can ameliorate this toxicity by regulating spermatogenesis and reducing oxidative stress.

Our results align with established evidence of triazole fungicide-induced reproductive toxicity^27,28^. Tebuconazole, a representative triazole fungicide, induces reproductive toxicity by impairing sperm motility, disrupting acrosome integrity, reducing intracellular Ca²⁺, and increasing lipid peroxidation^29^. Consistent with this mechanistic profile, PTC and dPTC exposure in male mammals elicited significant reproductive toxicity, manifested as reduced sperm counts and histopathological testicular damage. RNA-Seq analysis provided molecular corroboration, revealing significant enrichment of reproductive pathways including spermatogenesis, reproductive process, and spermatid development. These transcriptomic alterations directly underpin the observed phenotypic deficits in spermatogenesis and sperm quality, establishing a comprehensive mechanistic link between triazole fungicide exposure and male reproductive impairment. Spermatogenesis is tightly regulated by HPG axis hormones. LH and FSH stimulate Leydig and Sertoli cells, respectively, to establish a suitable hormonal milieu and provide androgen substrates essential for spermatogenesis^30,31^. We observed that PTC/dPTC exposure reduced LH, T, and DHT levels, while melatonin supplementation significantly restored LH, T, and DHT but did not significantly improve FSH or GnRH. These findings align with evidence that environmental pollutants—including fluorine and difenoconazole—disrupt reproductive function via HPG axis interference^32,33^. Melatonin’s well-documented protective effects against reproductive toxicity^34,35^ were further validated in this study, as it normalized PTC/dPTC-induced HPG axis dysregulation. Notably, melatonin reversed the downregulation of KISS1 and GPR54 expression in the hypothalamus, a key regulatory system where KISS1 activates GPR54 to modulate GnRH secretion and reproductive function^36,37^. We observed that melatonin supplementation reversed the PTC/dPTC-induced downregulation of KISS1/GPR54 expression. Melatonin has been shown to induce *Kiss1* gene expression, promote gonad maturation, and improve developmental capacity^38,39^, which aligns with our findings. These results suggest that melatonin may restore normal HPG axis function and improve spermatogenesis by upregulating KISS1/GPR54 expression following PTC/dPTC exposure, with the primary mechanism involving LH-mediated androgen restoration.

Furthermore, we observed that PTC and dPTC exposure induced abnormal expression of steroidogenic genes, which was normalized by melatonin supplementation. These findings align with prior evidence demonstrating that triazole fungicides disrupt steroidogenesis in reproductive tissues^40^. Notably, PTC/dPTC exposure upregulated *Cyp11a1, Cyp17a1, 3β-HSD*, and *17β-HSD* mRNA expression, yet resulted in a marked decrease in the corresponding protein content (e.g., CYP17A1, 17β-HSD3) and the precursor PREG, leading to impaired steroidogenesis. This clear transcriptional-translational decoupling, alongside StAR downregulation at both mRNA and protein levels, strongly indicates multi-layered disruption of the steroidogenic pathway. StAR is essential for cholesterol transport into mitochondria, initiating steroidogenesis^41^. The concurrent suppression of StAR likely creates a bottleneck, limiting cholesterol substrate availability for pregnenolone synthesis and contributing to the hormone deficit. Notably, melatonin effectively restored both the gene expression and the protein levels of these key factors. Previous studies confirm that melatonin promotes testosterone synthesis by upregulating *StAR* mRNA expression^42,43^, consistent with our observation that melatonin restored StAR levels and rescued steroidogenic function^23^. More importantly, our study extends this understanding by demonstrating that melatonin also rectifies the post-transcriptional deficits and rescues the entire steroidogenic cascade, thereby re-establishing functional hormone output. The normalization of steroidogenic gene and protein expression following melatonin treatment supports the conclusion that triazole fungicides disrupt testosterone homeostasis through impaired steroidogenesis, with melatonin alleviating toxicity by re-establishing functional steroidogenesis via StAR-mediated cholesterol transport. Our data suggest that melatonin’s rescue effect operates at least in part by re-establishing StAR-mediated cholesterol transport and mitigating the translational suppression of downstream enzymes. While these results clarify melatonin’s role in rescuing steroidogenesis, the precise molecular mechanisms underlying PTC/dPTC-induced post-transcriptional enzyme inhibition, such as alterations in protein stability, translational efficiency, or microRNA regulation, remain to be fully elucidated.

Oxidative stress is another important contributor to reproductive toxicity. Maintaining redox homeostasis is crucial for reproductive function, as oxidative damage from excessive ROS can negatively impact all stages of this process^44,45^. Triazole fungicides are recognized as oxidative stressors that induce oxidative damage in reproductive tissues^46,47^. Prior studies demonstrate that these compounds elevate ROS levels, modulate the activities of antioxidant enzymes (catalase, SOD), and contribute to male reproductive dysfunction^48^. Consistent with this mechanistic framework, our data reveal significant alterations in testicular oxidative stress markers following PTC/dPTC exposure, including elevated MDA, SOD, and GSH levels. Furthermore, two oxidative stress-related pathways were significantly enriched in the testicular RNA-Seq data. These results indicate that PTC and dPTC induce testicular oxidative stress. Melatonin, a potent antioxidant, has a protective role against testicular damage caused by exposure to various environmental pollutants^49^. In this study, melatonin supplementation effectively mitigated the PTC/dPTC-induced changes in testicular MDA, SOD, and GSH levels. This restoration of oxidative stress markers suggests that melatonin alleviates testicular oxidative stress and improves reproductive function. Melatonin exerts antioxidant effects by regulating the activity and expression of antioxidant enzymes in the testes^50^. Consistent with our results, previous studies have demonstrated that melatonin modulates the levels of GSH, SOD, MDA, catalase, and hydrogen peroxide, reducing oxidative stress and mitigating the reproductive toxicity of bisphenol A^51^. Melatonin also protects against reproductive damage by inhibiting downstream oxidative stress and apoptosis signals induced by glyphosate-based herbicides^52^. Various mechanisms underlying melatonin’s antioxidant effects have been elucidated^53,54^. However, the precise mechanisms through which melatonin confers its antioxidant actions in the context of PTC/dPTC-induced oxidative stress require further investigation.

The BTB maintains a highly stable microenvironment essential for germ cell survival and spermatogenesis. Testicular oxidative stress compromises BTB integrity, thereby impairing reproductive function^55^. The impact of triazole fungicides on BTB integrity remains unreported. Here, tight junction proteins OCCLUDIN (assessed via IHC) and ZO-1 (assessed via IF) were evaluated. PTC/dPTC exposure significantly reduced OCCLUDIN (IHC) and ZO-1 (IF) protein expression in testicular tissues, indicating BTB disruption, while melatonin supplementation restored OCCLUDIN and ZO-1 expression to basal levels, preserving BTB integrity. This aligns with prior evidence demonstrating melatonin’s protective role against BTB disruption^56^. Consistent with our results, melatonin reversed BTB integrity impairment induced by methamphetamine^57^and counteracted cadmium-induced BTB toxicity^58^.

Mechanistically, our data reveal significant activation of the MAPK/ERK signaling pathway following PTC/dPTC exposure, consistent with bromuconazole-induced MAPK dysregulation^59^. Melatonin supplementation reversed this overactivation, aligning with its established role in attenuating oxidative stress through MAPK/ERK modulation^60,61^. Notably, while Cao et al. reported melatonin-mediated p-ERK/ERK activation protecting against glyphosate-based herbicide (BSH)-induced apoptosis^52^, our study demonstrates that melatonin inhibits PTC/dPTC-triggered MAPK/ERK overactivation. This discrepancy likely reflects differential pathway responses: PTC/dPTC exposure activated MAPK/ERK, inducing oxidative stress and BTB disruption, whereas BSH inhibited ERK signaling. Oxidative stress compromises BTB integrity via MAPK-mediated mechanisms, including enhanced tight-junction protein ubiquitination, protease degradation, and endocytosis^62–64^. Given that PTC/dPTC-induced MAPK/ERK overactivation directly damages the BTB, melatonin’s protective effect manifests as inhibition of this pathological pathway. Consequently, melatonin’s protective effect against PTC/dPTC-induced BTB damage stems from targeted inhibition of this pathological MAPK/ERK activation, underscoring the context-dependent nature of melatonin’s regulatory actions on signaling pathways and necessitating exposure-specific mechanistic investigation.

Several limitations of this study should be acknowledged. First, while our study demonstrates melatonin’s protective effects against PTC/dPTC-induced toxicity, the absence of dose-response and time-course analyses limits our ability to precisely define the relationship between exposure, toxicity, and amelioration. Future studies with multiple doses and serial time-point measurements will be essential to fully characterize these dynamics. Second, although we observed significant alterations in testicular histopathology, hormone levels, and spermatogenic gene expression following PTC and dPTC exposure, we did not assess sperm motility or other functional parameters of sperm quality (e.g., concentration, morphology, or viability). Sperm motility is a critical determinant of male fertility, and its evaluation would have provided more direct evidence of functional reproductive impairment. The absence of sperm functional data limits the comprehensiveness of our assessment of male fertility outcomes. Another potential limitation of this study is that while we observed upregulation of key steroidogenic genes (*Cyp11a1, Cyp17a1, 3β-HSD, 17β-HSD*) in PTC/dPTC-exposed mice, their enzymatic activities were not directly measured. Our follow-up ELISA analysis confirmed a decrease in the protein content of key factors (StAR, PREG, CYP17A1, 17β-HSD3), providing direct evidence for post-transcriptional suppression. The clear discordance between increased mRNA expression, decreased protein levels, and reduced androgen output strongly supports the occurrence of post-translational inhibition or functional impairment within the steroidogenic pathway. However, the precise nature of this inhibition—whether due to reduced protein stability, impaired catalytic activity, or other mechanisms—remains to be determined. Without direct assessment of enzyme activities (e.g., Cyp11a1, 17β-HSD) or cholesterol transport efficiency mediated by StAR, a complete mechanistic understanding of the functional blockade is still evolving. Additionally, the compensatory upregulation of steroidogenic genes in the context of suppressed HPG axis signaling highlights a complex regulatory interplay that warrants further investigation. Future studies employing direct enzyme activity assays, substrate-tracing approaches, and investigations into protein turnover will be essential to fully elucidate the molecular mechanisms by which PTC and dPTC disrupt testicular steroidogenesis at a functional level.

In conclusion, this study revealed the reproductive toxicity of PTC and dPTC to male mammals. Melatonin can ameliorate the reproductive toxicity of PTC and dPTC by regulating the secretion of HPG axis hormones, improving spermatogenesis, regulating oxidative stress, and protecting the integrity of BTB. Our data support the conclusion that PTC and dPTC are reproductively toxic in mammals, and imply that melatonin might serve as a potential therapeutic option for reducing the adverse reproductive effects of triazole fungicides.

## Materials and methods

### Reagents and antibodies

PTC (CAS No: 178928-70-6) and dPTC (CAS No: 120983-64-4) were obtained from Sigma-Aldrich (St. Louis, MO, USA). Melatonin (CAS No: 73-31-4, purity ≥98%) was purchased from Macklin (Shanghai, China). Corn oil and anhydrous ethanol were also purchased from Macklin (Shanghai, China). ELISA kits were purchased from Jiangsu Meibiao Biological Technology Co., Ltd. (Xuzhou, Jiangsu, China). Antibodies were obtained from Abcam (Cambridge, UK), Life Technologies (Carlsbad, CA, USA), Proteintech (Wuhan, Hubei, China), and ABclonal (Wuhan, Hubei, China). TRIzol reagent, SYBR Green Master Mixes, and secondary antibody (Goat anti-Rabbit IgG (H + L)) were purchased from Thermo Fisher Scientific (Waltham, MA, USA). StarScript III kits and QuickBlock™ Blocking Buffer were obtained from GenStar (Beijing, China) and Beyotime Biotechnology (Shanghai, China), respectively. The QIAGEN RNeasy Mini Kit was purchased from QIAGEN (Hilden, Germany).

### Animals and treatment

All procedures involving animals and their care were reviewed and approved by the Medical Ethics Committee of Southern Medical University (Approval Code: SMUL202405018), in accordance with institutional guidelines. A total of 48 adult male C57BL/6 mice (8–9 weeks old) were used in this study. All mice were housed under specific pathogen-free laboratory conditions with diet and water ad libitum. PTC and dPTC were dissolved in corn oil. Melatonin was dissolved in ethanol and then diluted in normal saline to a final ethanol concentration of 5%.

Male mice were randomly divided into three groups, eight in each group: the PTC group was intragastrically administrated with PTC at a dose of 5 mg/kg/day; the dPTC group was intragastrically administrated with dPTC at a dose of 1 mg/kg/day; and the control group was intragastrically administered an equal volume of corn oil. The entire experiment lasted for 30 days. The doses were selected according to previously reported studies and are equivalent to the initial residues of PTC and dPTC in cereals or wheat^65–67^. For the melatonin supplementation experiment, the mice were randomly divided into three groups, eight in each group: the PTC + MT group, the dPTC+MT group, and the Oil+MT group. The PTC and dPTC exposure models were established as described above. At the start of the modeling period, mice were intraperitoneally injected with melatonin at a dose of 10 mg/kg/day, concurrently with exposure to PTC or dPTC. Melatonin treatment continued until the end of the experiment. This dose of melatonin has been demonstrated to exert reproductive protective effects in previous studies^22,68^. After the mice were euthanized, serum, hypothalamus, and testes were collected and stored at −80 °C for subsequent analysis.

### Assessment of the sperm count

The sperm suspensions were prepared to measure the sperm count according to our previous research^69^. Briefly, the cauda epididymis from each side was dissected, and both tissues were transferred into 1 mL of pre-warmed phosphate-buffered saline (PBS) maintained at 37 °C. The tissues were finely minced and incubated for 5 min to facilitate complete sperm release into the medium. Spermatozoa were gently dispersed by mild pipetting to ensure a homogeneous suspension. Total sperm count was performed using a Neubauer hemocytometer under light microscopy, with duplicate counts conducted for each sample to ensure accuracy. The total sperm number was expressed as the sum from both cauda epididymides per mouse.

### Histopathological analysis

Following our established protocol^70^, testicular tissues were fixed with 4% paraformaldehyde, washed with PBS, dehydrated through a graded alcohol series, cleared in xylene, and embedded in paraffin blocks. Sections (5 μm thick) were cut, deparaffinized, rehydrated, and stained with H&E. Images were captured using a Leica DMI 3000 microscope (Leica Microsystems, Wetzlar, Germany). The area of the seminiferous tubules, the diameter of the seminiferous tubules, and the number of spermatocytes per tubule were analyzed using ImageJ software.

### Measurement of the HPG axis hormone secretion

Whole blood was allowed to clot at room temperature for 30 min, followed by centrifugation at 3000 rpm (4 °C, 10 min) to obtain serum. According to the manufacturer’s instructions (Jiangsu Meibiao Biological Technology Co., Ltd., Jiangsu, China), ELISA kits were used to quantify the hormones of the HPG axis, including serum testosterone (T, Cat #MB-3305B), dihydrotestosterone (DHT, Cat #MB-3267B), androstenedione (ASD, Cat #MB-3307B), LH (Cat #MB-3318B), FSH (Cat #MB-3291B) and GnRH (Cat #MB-3242B).

### Immunohistochemistry (IHC) and immunofluorescence (IF) staining

For IHC, testicular tissues were fixed with 4% paraformaldehyde, permeabilized with 0.01% saponin, and blocked in 1% BSA before overnight incubation with primary antibodies (Occludin, 1:100, Abcam, Cat #ab216327) at 4 °C. The sections were then incubated with secondary antibodies (Donkey anti-Rabbit IgG (H + L), 1:400, Life Technologies, Cat #A21207) at room temperature for 1 h. Images were taken with a laser confocal microscope, and the percentage of Occludin-positive area (positive area/total tissue area) was analyzed by ImageJ. IF staining analysis was conducted as described in a previous publication^69^. Briefly, testicular tissues were fixed in 4% paraformaldehyde, followed by sectioning, antigen retrieval, and antigen blocking. The sections were incubated with primary antibodies (ZO-1, 1:500, Abcam, Cat #ab221547) overnight at 4 °C. Secondary antibody (Donkey anti-Rabbit IgG (H + L), 1:400, Life Technologies, Cat #A21207) incubation and DAPI staining (Solarbio, Cat #C0060) followed. A laser confocal microscope (Leica TCS SP8, Leica Microsystems, Wetzlar, Germany) was used for observation, and ImageJ was used for mean fluorescence intensity (total fluorescence intensity/total fluorescence area) analysis.

### Measurement of oxidative stress levels

To assess oxidative stress, levels of malondialdehyde (MDA), superoxide dismutase (SOD), and glutathione (GSH) in testicular tissues were measured using ELISA kits as per the manufacturer’s guidelines. MDA (Cat #MB-5892A), SOD (Cat #MB-3125A), and GSH (Cat #MB-6324A) were obtained from Jiangsu Meibiao Biological Technology Co., Ltd. (Jiangsu, China).

### Measurement of protein content of key steroidogenic factors

To quantify the testicular protein content of key steroidogenic factors (StAR, pregnenolone [PREG], CYP17A1, and 17β-HSD3), ELISA kits were used for detectionas in accordance with the instructions. StAR (Cat # MM-45301M1), PREG (Cat # MM-928606O1), CYP17A1 (Cat #MM-48956M1), and 17β-HSD3 (Cat #MM-49539M1) were obtained from Jiangsu Meimian Industrial Co., Ltd. (Jiangsu, China).

### RT-qPCR analysis

As described previously^70^, total RNA was isolated from testicular and hypothalamic tissues via TRIzol reagent, and its quality and concentration were measured using a NanoDrop spectrometer. Subsequently, StarScript III kits (GenStar, Beijing, China; Cat #A230-10) were used to synthesize the complementary DNA (cDNA), and qPCR was performed via SYBR Green Master Mixes (GenStar, Beijing, China, Cat #A301-10) on an ABI Q6 Flex (Applied Biosystems, USA). Relative gene expression was determined via the 2^−△△CT^ method and normalized against *β-Actin*. Detailed primer sequences are listed in Supplementary Information 1.

### Western blot analysis

As described previously^71^, proteins from testicular tissues were extracted via RIPA buffer (Beyotime Biotechnology, Jiangsu, China) supplemented with 1% PMSF and 1% phosphatase inhibitor. The protein concentration was then measured via a BCA protein assay kit (Thermo Fisher Scientific, USA). Equal amounts of protein were resolved by 8–12% SDS-PAGE, transferred to PVDF membranes, and blocked with QuickBlock™ Blocking Buffer (Beyotime). Overnight incubation of the membranes was performed at 4 °C using primary antibodies, followed by a 1.5-h incubation with secondary antibodies at room temperature. Protein bands were visualized using a Tanon Gel Image System (version 4.2) and quantified with ImageJ, with normalization to β-actin. The following primary antibodies were employed: (a) GAPDH (1:1000, Proteintech, Cat # 80570-1-RR); (b) p38 MAPK (1:1000, Proteintech, Cat #14064-1-AP); (c) phospho-p38 MAPK (Thr180/Tyr182) (1:1000, Proteintech, Cat #28796-1-AP); (d) ERK1/2 (1:1000, ABclonal, Cat #A4782); and (e) phospho-ERK1/2 (Thr202/Tyr204) (1:1000, Proteintech, Cat #28733-1-AP).

### RNA sequencing (RNA-seq)

In line with our prior research, total RNA was extracted from testicular tissues using the QIAGEN RNeasy Mini Kit (Cat #74104). Majorbio Bio-Pharm Biotechnology (Shanghai, China) performed all sequencing analyses, which strictly adhered to the manufacturer’s protocols (Illumina, San Diego, CA). Data analysis was conducted on the Majorbio Cloud platform, where DEGs were identified using the criteria of FDR < 0.05 and |log2FC| ≥ 1.5. Further analyses, such as volcano plot analysis, Venn analysis, and GO functional enrichment, were also carried out on the Majorbio Cloud platform^72,73^.

### Statistics and reproducibility

All data are expressed as mean ± standard error of the mean (SEM). Each experiment was performed with a minimum of three biological replicates and three technical replicates. Replicates refer to the complete repetitions of the entire detection process, including sample pretreatment, detection steps and procedures, and endpoint measurement. Data normality and homoscedasticity were assessed using the Shapiro-Wilk test and the Brown–Forsythe test (a robust variant of Levene’s test), respectively (Supplementary Data 1). All datasets met the normality assumption (*P* > 0.05). For the vast majority of parameters confirming homoscedasticity (*P* > 0.05), a standard one-way ANOVA was performed. For the few parameters where the Brown–Forsythe test indicated heterogeneity of variance, Welch’s ANOVA was used for group comparisons. For three-group comparisons under a single factor, where the planned analysis focused on two pre-specified comparisons of each treatment versus the Oil control, one-way ANOVA followed by Holm-Sidak multiple comparisons test was used. This method provides optimal power for controlling the family-wise error rate (FWER) in a small set of planned comparisons. For analyses involving six groups, which involved all possible pairwise comparisons, we used Tukey’s post hoc test following a significant one-way ANOVA. This is the standard method for FWER control in unplanned, all-pairwise comparisons. Statistical significance was defined as *P* < 0.05 (*P < 0.05, **P < 0.01, ***P < 0.001, *N.S*. non-significant (*P* > 0.05)). The source data behind the graphs in the paper is shown in the Supplementary Data 2. All analyses were performed using GraphPad Prism 9.0.

### Ethics approval and consent to participate

All animal care and experimental protocols were approved by the Medical Ethics Committee of Southern Medical University (Approval Code: SMUL202405018).

### Reporting summary

Further information on research design is available in the Nature Portfolio Reporting Summary linked to this article.

## Supplementary information

Supplementary Information

Supplementary Data 1

Supplementary Data 2

Supplementary Data 3

Description of Additional Supplementary Files

Reporting Summary

## Data Availability

The RNA sequencing datasets from the testis samples have been stored in the NCBI Sequence Read Archive (SRA) and are available under the accession number PRJNA1185821.
