# Supplementary material for: Melatonin attenuates reproductive toxicity of prothioconazole and its metabolite via spermatogenesis and oxidative stress
Source: Commun Biol. 2026 May 23;9:1105. doi: 10.1038/s42003-026-10311-y (PMC13478280; doi:10.1038/s42003-026-10311-y)
Supplement: Supplementary file 1 [file 42003_2026_10311_MOESM1_ESM.pdf]

Supplementary Table 1. The primers used in the present study.

GeneSymbol	Forward/Reverse	Primer（5‘ to 3’）
Kiss1	Forward	CTCCTCTGTGTCGCCACCTA
	Reverse	TTCCCAGGCATTAACGAGTTC
Gpr54	Forward	CCGTCCAACGCTTCAGGAT
	Reverse	GTGTAGCGAAAAACAGGGGAA
Srd5a1	Forward	GAGTTGGATGAGTTGCGCCTA
	Reverse	GGACCACTGCGAGGAGTAG
StAR	Forward	ATGTTCCTCGCTACGTTCAAG
	Reverse	CCCAGTGCTCTCCAGTTGAG
Cyp11a1	Forward	AGGTCCTTCAATGAGATCCCTT
	Reverse	TCCCTGTAAATGGGGCCATAC
Cyp17a1	Forward	GCCCAAGTCAAAGACACCTAAT
	Reverse	GTACCCAGGCGAAGAGAATAGA
3β-HSD	Forward	TGGACAAAGTATTCCGACCAGA
	Reverse	GGCACACTTGCTTGAACACAG
17β-HSD	Forward	ACTTGGCTGTTCGCCTAGC
	Reverse	GAGGGCATCCTTGAGTCCTG
Gja1	Forward	ACAGCGGTTGAGTCAGCTTG
	Reverse	GAGAGATGGGGAAGGACTTGT
Cdh2	Forward	AGCGCAGTCTTACCGAAGG
	Reverse	TCGCTGCTTTCATACTGAACTTT
Occludin	Forward	CCGTCCAACGCTTCAGGAT
	Reverse	GTGTAGCGAAAAACAGGGGAA
β-Actin	Forward	GAGTTGGATGAGTTGCGCCTA
	Reverse	GGACCACTGCGAGGAGTAG
