# Supplementary material for: Melatonin attenuates reproductive toxicity of prothioconazole and its metabolite via spermatogenesis and oxidative stress
Source: Commun Biol. 2026 May 23;9:1105. doi: 10.1038/s42003-026-10311-y (PMC13478280; doi:10.1038/s42003-026-10311-y)
Supplement: Supplementary file 3 [file 42003_2026_10311_MOESM2_ESM.docx]

Normality test and homogeneity of variance test were assessed using the Shapiro-Wilk test and the Brown-Forsythe test (a robust variant of Levene’s test). The statistical results are as follow:

Body Weight Curve

The Shapiro-Wilk test (all P > 0.05)

Shapiro-Wilk test

W	0.9435	0.8578	0.9699	0.9141	0.8750	0.7955

P value	0.6873	0.1818	0.8915	0.4642	0.2470	0.0535

Passed normality test (alpha=0.05)?	Yes	Yes	Yes	Yes	Yes	Yes

P value summary	ns	ns	ns	ns	ns	ns

Sperm Count

The Shapiro-Wilk test (all P > 0.05) and the homogeneity of variance test (Brown-Forsythe test, P = 0.5394)

Shapiro-Wilk test

W	0.9508	0.8883	0.9427	0.9600	0.9383	0.8988

P value	0.7372	0.2659	0.6635	0.8188	0.6231	0.3239

Passed normality test (alpha=0.05)?	Yes	Yes	Yes	Yes	Yes	Yes

P value summary	ns	ns	ns	ns	ns	ns

Testicle / Weight

The Shapiro-Wilk test (all P > 0.05) and the homogeneity of variance test (Brown-Forsythe test, P = 0.1477)

Shapiro-Wilk test

W	0.9466	0.9452	0.9312	0.9748	0.8978	0.9449

P value	0.6991	0.6859	0.5609	0.9310	0.3182	0.6829

Passed normality test (alpha=0.05)?	Yes	Yes	Yes	Yes	Yes	Yes

P value summary	ns	ns	ns	ns	ns	ns

Spermatocytes number / tubule

The Shapiro-Wilk test (all P > 0.05) and the homogeneity of variance test (Brown-Forsythe test, P = 0.8362)

Shapiro-Wilk test

W	0.9417	0.9685	0.9747	0.9234	0.9551	0.9740

P value	0.1957	0.6543	0.7984	0.0787	0.3724	0.7832

Passed normality test (alpha=0.05)?	Yes	Yes	Yes	Yes	Yes	Yes

P value summary	ns	ns	ns	ns	ns	ns

Diameter of seminiferous tubule

The Shapiro-Wilk test (all P > 0.05) and the homogeneity of variance test (Brown-Forsythe test, P = 0.4348)

Shapiro-Wilk test

W	0.8793	0.9332	0.9063	0.8618	0.9028	0.9640

P value	0.1282	0.4802	0.2567	0.0801	0.2351	0.8302

Passed normality test (alpha=0.05)?	Yes	Yes	Yes	Yes	Yes	Yes

P value summary	ns	ns	ns	ns	ns	ns

Area of seminiferous tubule

The Shapiro-Wilk test (all P > 0.05) and the homogeneity of variance test (Brown-Forsythe test, P = 0.3619)

Shapiro-Wilk test

W	0.9656	0.9399	0.9629	0.9366	0.9610	0.9731

P value	0.3688	0.0673	0.3122	0.0541	0.2756	0.5691

Passed normality test (alpha=0.05)?	Yes	Yes	Yes	Yes	Yes	Yes

T-ELISA

The Shapiro-Wilk test (all P > 0.05) and the homogeneity of variance test (Brown-Forsythe test, P = 0.0709)

Shapiro-Wilk test

W	0.9841	0.9163	0.8889	0.9942	0.9133	0.9301

P value	0.9803	0.4004	0.2288	0.9992	0.3779	0.5168

Passed normality test (alpha=0.05)?	Yes	Yes	Yes	Yes	Yes	Yes

P value summary	ns	ns	ns	ns	ns	ns

DHT-ELISA

The Shapiro-Wilk test (all P > 0.05) and the homogeneity of variance test (Brown-Forsythe test, P = 0.5278)

Shapiro-Wilk test

W	0.8880	0.9691	0.9012	0.9209	0.9759	0.8499

P value	0.2244	0.8906	0.2965	0.4375	0.9399	0.0951

Passed normality test (alpha=0.05)?	Yes	Yes	Yes	Yes	Yes	Yes

P value summary	ns	ns	ns	ns	ns	ns

ASD-ELISA

The Shapiro-Wilk test (all P > 0.05), but the homogeneity of variance test showed heterogeneous variances (Brown-Forsythe test, P = 0.0366)

Shapiro-Wilk test

W	0.9118	0.9641	0.9166	0.9775	0.9537	0.9357

P value	0.3669	0.8479	0.4028	0.9496	0.7488	0.5691

Passed normality test (alpha=0.05)?	Yes	Yes	Yes	Yes	Yes	Yes

P value summary	ns	ns	ns	ns	ns	ns

FSH-ELISA

The Shapiro-Wilk test (all P > 0.05), but the homogeneity of variance test showed heterogeneous variances (Brown-Forsythe test, P = 0.0221)

Shapiro-Wilk test

W	0.9480	0.9038	0.9150	0.8745	0.8331	0.9640

P value	0.6906	0.3123	0.3908	0.1667	0.0640	0.8473

Passed normality test (alpha=0.05)?	Yes	Yes	Yes	Yes	Yes	Yes

P value summary	ns	ns	ns	ns	ns	ns

LH-ELISA

The Shapiro-Wilk test (all P > 0.05) and the homogeneity of variance test (Brown-Forsythe test, P = 0.2516)

Shapiro-Wilk test

W	0.9138	0.9151	0.9261	0.9811	0.9385	0.9100

P value	0.3812	0.3912	0.4816	0.9683	0.5961	0.3539

Passed normality test (alpha=0.05)?	Yes	Yes	Yes	Yes	Yes	Yes

P value summary	ns	ns	ns	ns	ns	ns

GnRH-ELISA

The D'Agostino & Pearson test	(all P > 0.05) and the homogeneity of variance test (Brown-Forsythe test, P = 0.0959)

D'Agostino & Pearson test

K2	4.050	0.6343	1.345	3.668	0.5952	2.040

P value	0.1320	0.7282	0.5105	0.1598	0.7426	0.3606

Passed normality test (alpha=0.05)?	Yes	Yes	Yes	Yes	Yes	Yes

P value summary	ns	ns	ns	ns	ns	ns

KISS1-RT-qPCR

The Shapiro-Wilk test (all P > 0.05) and the homogeneity of variance test (Brown-Forsythe test, P = 0.4455)

Shapiro-Wilk test

W	0.9479	0.9037	0.9517	0.8975	0.9551	0.8758

P value	0.5600	0.3973	0.5766	0.3775	0.5922	0.3123

Passed normality test (alpha=0.05)?	Yes	Yes	Yes	Yes	Yes	Yes

P value summary	ns	ns	ns	ns	ns	ns

GPR45-RT-qPCR

The Shapiro-Wilk test (all P > 0.05) and the homogeneity of variance test (Brown-Forsythe test, P = 0.7754)

Shapiro-Wilk test

W	0.8837	0.9972	0.8592	0.9997	0.8023	0.8632

P value	0.3353	0.8982	0.2655	0.9686	0.1200	0.2764

Passed normality test (alpha=0.05)?	Yes	Yes	Yes	Yes	Yes	Yes

P value summary	ns	ns	ns	ns	ns	ns

SRD5A1-RT-qPCR

The Shapiro-Wilk test (all P > 0.05) and the homogeneity of variance test (Brown-Forsythe test, P = 0.4108)

Shapiro-Wilk test

W	0.8618	0.8919	0.8376	1.000	0.9576	0.9690

P value	0.2724	0.3602	0.2078	0.9894	0.6039	0.6617

Passed normality test (alpha=0.05)?	Yes	Yes	Yes	Yes	Yes	Yes

P value summary	ns	ns	ns	ns	ns	ns

StAR-RT-qPCR

The Shapiro-Wilk test (all P > 0.05) and the homogeneity of variance test (Brown-Forsythe test, P = 0.3351)

Shapiro-Wilk test

W	0.9998	0.9447	0.9262	0.9463	0.8935	0.9325

P value	0.9750	0.5466	0.4747	0.5535	0.3650	0.4982

Passed normality test (alpha=0.05)?	Yes	Yes	Yes	Yes	Yes	Yes

P value summary	ns	ns	ns	ns	ns	ns

CYP11A1-RT-qPCR

The Shapiro-Wilk test (all P > 0.05) and the homogeneity of variance test (Brown-Forsythe test, P = 0.8822)

Shapiro-Wilk test

W	0.9923	0.7881	0.9684	0.8231	0.8257	0.9818

P value	0.8323	0.0863	0.6588	0.1709	0.1775	0.7412

Passed normality test (alpha=0.05)?	Yes	Yes	Yes	Yes	Yes	Yes

P value summary	ns	ns	ns	ns	ns	ns

CYP17A1-RT-qPCR

The Shapiro-Wilk test (all P > 0.05) and the homogeneity of variance test (Brown-Forsythe test, P = 0.2361)

Shapiro-Wilk test

W	0.7838	0.7733	0.9883	0.9620	0.7879	0.8357

P value	0.0764	0.0523	0.7931	0.6254	0.0859	0.2029

Passed normality test (alpha=0.05)?	Yes	Yes	Yes	Yes	Yes	Yes

P value summary	ns	ns	ns	ns	ns	ns

3β-HSD-RT-qPCR

The Shapiro-Wilk test (all P > 0.05) and the homogeneity of variance test (Brown-Forsythe test, P = 0.8349)

Shapiro-Wilk test

W	0.9129	0.9948	0.9047	0.8490	0.7745	0.9020

P value	0.4279	0.8627	0.4007	0.2378	0.0551	0.3920

Passed normality test (alpha=0.05)?	Yes	Yes	Yes	Yes	Yes	Yes

P value summary	ns	ns	ns	ns	ns	ns

17β-HSD-RT-qPCR

The Shapiro-Wilk test (all P > 0.05) and the homogeneity of variance test (Brown-Forsythe test, P = 0.3912)

Shapiro-Wilk test

W	0.8226	0.9998	0.9469	0.8947	0.9694	0.9898

P value	0.1698	0.9745	0.5559	0.3689	0.6644	0.8069

Passed normality test (alpha=0.05)?	Yes	Yes	Yes	Yes	Yes	Yes

P value summary	ns	ns	ns	ns	ns	ns

MDA-ELISA

The Shapiro-Wilk test (all P > 0.05) and the homogeneity of variance test (Brown-Forsythe test, P = 0.9224)

Shapiro-Wilk test

W	0.8973	0.9809	0.8386	0.8872	0.9010	0.8423

P value	0.3150	0.9640	0.0963	0.2603	0.3371	0.1044

Passed normality test (alpha=0.05)?	Yes	Yes	Yes	Yes	Yes	Yes

P value summary	ns	ns	ns	ns	ns	ns

GSH-ELISA

The Shapiro-Wilk test (all P > 0.05) and the homogeneity of variance test (Brown-Forsythe test, P = 0.6632)

Shapiro-Wilk test

W	0.8598	0.9104	0.8295	0.9090	0.9021	0.8313

P value	0.1508	0.3986	0.0792	0.3889	0.3438	0.0824

Passed normality test (alpha=0.05)?	Yes	Yes	Yes	Yes	Yes	Yes

P value summary	ns	ns	ns	ns	ns	ns

SOD-ELISA

The Shapiro-Wilk test (all P > 0.05) and the homogeneity of variance test (Brown-Forsythe test, P = 0.4665)

Shapiro-Wilk test

W	0.9502	0.9304	0.8481	0.9214	0.9622	0.9481

P value	0.7313	0.5545	0.1181	0.4805	0.8370	0.7128

Passed normality test (alpha=0.05)?	Yes	Yes	Yes	Yes	Yes	Yes

P value summary	ns	ns	ns	ns	ns	ns

OCC IHC

The Shapiro-Wilk test (all P > 0.05), but the homogeneity of variance test showed heterogeneous variances (Brown-Forsythe test, P = 0.0026)

W	0.9512	0.8642	0.8640

P value	0.6545	0.0553	0.0550

Passed normality test (alpha=0.05)?	Yes	Yes	Yes

P value summary	ns	ns	ns

ZO-1 IF

The Shapiro-Wilk test (all P > 0.05) and the homogeneity of variance test (Brown-Forsythe test, P = 0.1870)

Shapiro-Wilk test

W	0.8938	0.9331	0.8983	0.9305	0.8705	0.9089

P value	0.2183	0.5117	0.2422	0.4863	0.1246	0.3082

Passed normality test (alpha=0.05)?	Yes	Yes	Yes	Yes	Yes	Yes

P value summary	ns	ns	ns	ns	ns	ns

Gja1-RT-qPCR

The Shapiro-Wilk test (all P > 0.05) and the homogeneity of variance test (Brown-Forsythe test, P = 0.6762)

Shapiro-Wilk test

W	0.9998	0.9396	0.9996	0.9851	0.8353	0.9921

P value	0.9760	0.5259	0.9641	0.7666	0.2020	0.8302

Passed normality test (alpha=0.05)?	Yes	Yes	Yes	Yes	Yes	Yes

P value summary	ns	ns	ns	ns	ns	ns

Cdh2-RT-qPCR

The Shapiro-Wilk test (all P > 0.05) and the homogeneity of variance test (Brown-Forsythe test, P = 0.9553)

Shapiro-Wilk test

W	0.9559	0.8882	0.8552	0.9274	0.9411	0.9400

P value	0.5960	0.3490	0.2545	0.4788	0.5319	0.5273

Passed normality test (alpha=0.05)?	Yes	Yes	Yes	Yes	Yes	Yes

P value summary	ns	ns	ns	ns	ns	ns

PREG-ELISA

The Shapiro-Wilk test (all P > 0.05) and the homogeneity of variance test (Brown-Forsythe test, P = 0.6060)

Shapiro-Wilk test

W	0.9773	0.9722	0.9306	0.8591	0.9149	0.9651

P value	0.9455	0.9141	0.5563	0.1487	0.4311	0.8614

Passed normality test (alpha=0.05)?	Yes	Yes	Yes	Yes	Yes	Yes

P value summary	ns	ns	ns	ns	ns	ns

StAR-ELISA

The Shapiro-Wilk test (all P > 0.05) and the homogeneity of variance test (Brown-Forsythe test, P = 0.5892)

Shapiro-Wilk test

W	0.9342	0.8126	0.8161	0.9465	0.9705	0.9287

P value	0.5872	0.0545	0.0589	0.6975	0.9022	0.5400

Passed normality test (alpha=0.05)?	Yes	Yes	Yes	Yes	Yes	Yes

P value summary	ns	ns	ns	ns	ns	ns

CYP17A1-ELISA

The Shapiro-Wilk test (all P > 0.05) and the homogeneity of variance test (Brown-Forsythe test, P = 0.3148)

Shapiro-Wilk test

W	0.8692	0.9494	0.9023	0.8746	0.9092	0.8609

P value	0.1827	0.7245	0.3450	0.2034	0.3904	0.1542

Passed normality test (alpha=0.05)?	Yes	Yes	Yes	Yes	Yes	Yes

P value summary	ns	ns	ns	ns	ns	ns

17β-HSD3-ELISA

The Shapiro-Wilk test (all P > 0.05) and the homogeneity of variance test (Brown-Forsythe test, P = 0.3843)

Shapiro-Wilk test

W	0.9706	0.9014	0.9365	0.8347	0.8247	0.8952

P value	0.9028	0.3395	0.6075	0.0887	0.0712	0.3030

Passed normality test (alpha=0.05)?	Yes	Yes	Yes	Yes	Yes	Yes

P value summary	ns	ns	ns	ns	ns	ns
