# Supplementary material for: Melatonin attenuates reproductive toxicity of prothioconazole and its metabolite via spermatogenesis and oxidative stress
Source: Commun Biol. 2026 May 23;9:1105. doi: 10.1038/s42003-026-10311-y (PMC13478280; doi:10.1038/s42003-026-10311-y)
Supplement: Supplementary file 4 [file 42003_2026_10311_MOESM5_ESM.docx]

**Description of Additional Supplementary File**

File name: Supplementary Data 1
Description: The statistical results of the normality and homoscedasticity of the data

File name: Supplementary Data 2
Description: The original protein band

File name: Supplementary Data 3
Description: The source data behind the graphs in the paper
